# Novel Roles of the Greatwall Kinase Rim15 in Yeast Oxidative Stress Tolerance through Mediating Antioxidant Systems and Transcriptional Regulation

**DOI:** 10.3390/antiox13030260

**Published:** 2024-02-21

**Authors:** Xue-Qing Wang, Bing Yuan, Feng-Li Zhang, Chen-Guang Liu, Choowong Auesukaree, Xin-Qing Zhao

**Affiliations:** 1 State Key Laboratory of Microbial Metabolism, Joint International Research Laboratory of Metabolic & Developmental Sciences, School of Life Sciences and Biotechnology, Shanghai Jiao Tong University, Shanghai 200240, China; xueqingwang@sjtu.edu.cn (X.-Q.W.); bing-yuan@sjtu.edu.cn (B.Y.); zhangfengli@sjtu.edu.cn (F.-L.Z.); cg.liu@sjtu.edu.cn (C.-G.L.); 2 Department of Biotechnology, Faculty of Science, Mahidol University, Bangkok 10400, Thailand; choowong.aue@mahidol.ac.th; 3 Mahidol University-Osaka University Collaborative Research Center for Bioscience and Biotechnology, Faculty of Science, Mahidol University, Bangkok 10400, Thailand

**Keywords:** *Saccharomyces cerevisiae*, protein kinase Rim15, Yap1, acetic acid stress tolerance, oxidative stress tolerance, antioxidant systems, kinase activity

## Abstract

The Greatwall-family protein kinase Rim15 is associated with the nutrient starvation response, whereas its role in oxidative stress responses remains unclear. Here, acetic acid and peroxide were used as two oxidative stress elicitors. The antioxidant indicator assay under acetic acid stress revealed the impaired growth in *rim15*Δ related to the regulation of antioxidant systems. Comparative transcriptome analysis revealed that differentially expressed genes (DEGs) are predicted to be mostly regulated by oxidative stress-responsive transcriptional factor Yap1. Among the DEGs, acetic acid stress-induced genes were found, and *YAP1* disruption also inhibited their induction. The deletion of Rim15 or the Rim15 kinase domain in *yap1*Δ did not further decrease the gene expression, suggesting that Rim15 functions together with Yap1 in regulating acetic acid stress-induced genes, which requires Rim15 kinase activity. Additionally, Rim15 regulated H_2_O_2_ stress tolerance through partially similar but special mechanisms in that Rim15 kinase activity impacted acetic acid and H_2_O_2_ stress tolerance in different degrees, indicating the different mechanisms underlying Rim15-mediated redox regulation against different stressors. These results benefit the better understanding of stress signaling pathways related to Rim15. Given that Rim15 and some of its target genes are conserved across eukaryotes, these results also provide a basis for studies of oxidative stress-related processes in other organisms.

## 1. Introduction

Rim15 protein belongs to the Greatwall-family protein kinases and is conserved among eukaryotes, whose homologs are Ppk18 in fission yeast and Mastl in mammals [1]. In higher organisms, the Greatwall-family kinase plays important roles in the cell cycle, meiotic maturation, and cancer [1]. Budding yeast *Saccharomyces cerevisiae* has been widely used as a model system for studying eukaryotes, including humans. In budding yeast, the main role of Rim15 is nutrient starvation responses, being inhibited by some signaling pathways, such as PKA (Protein kinase A) and TORC1 (TOR complex I) [2]. During nitrogen starvation, Rim15 acts as an indirect transcriptional regulator via its function in the inactivation of transcriptional repressors Ume6 and Rph1, leading to the promotion of autophagy (ATG) gene expression [3,4]. ATG genes are also conserved in higher eukaryotes, and autophagy dysregulation is related to multiple human pathologies [5]. Furthermore, the deletion of *RIM15* leads to a defect in the chronological lifespan (CLS) [6]. Therefore, in-depth studies on Rim15 might also contribute to studies of human diseases and longevity. On the other hand, Rim15 can activate transcriptional factor Gis1 indirectly and transcriptional factors Hsf1, Msn2, and Msn4 through direct phosphorylation to regulate the genes required for survival under nutrient starvation [7,8]. This process is also regulated by the upstream pathways PKA and TORC1 [2]. Although Rim15 is well known to exert its function in nutrient stress, its role in oxidative stress remains largely unexplored.

Oxidative stress is generated by the imbalance between oxidants and antioxidants, mostly triggered by excessive reactive oxygen species (ROS) [9]. Not only oxidizing agents but also other environmental conditions such as nutrition starvation, high temperature, ultraviolet (UV) ray radiation, heavy metals, medicines, and some toxic inhibitors (formic acid, acetic acid, furfural, and 5-hydroxymethylfurfural) in lignocellulosic hydrolysate can also lead to ROS accumulation [10,11,12]. An appropriate redox state is critical for cell viability, growth, and proliferation [10]. The removal of excessive ROS to maintain the redox balance at an appropriate status is regulated by the antioxidant systems, which are divided into enzymatic and non-enzymatic antioxidants [9]. The major antioxidant enzymes are superoxide dismutase (SOD), catalase (CAT), and glutathione peroxidase (GPx), with a cascade reaction. H_2_O_2_ converted from superoxide anions (O_2_^−•^) by SOD is broken down into water and oxygen by CAT and GPx [13,14]. Furthermore, reduced glutathione (GSH) is the most important non-enzymatic antioxidant. Its antioxidant effects are achieved through the degradation of hydrogen peroxide and lipid peroxide [15]. Moreover, oxidative stress is well known to be related to aging, inflammation, and diseases in mammalian cells [11,16,17]. Therefore, studies on oxidative stress tolerance provide a deeper understanding of not only the acetic acid stress response but also common mechanisms involved in responding to different stresses, which would give more insights into relevant human diseases.

Oxidative stress can also be induced by acetic acid. In budding yeast, acetic acid is one of the byproducts of ethanol fermentation. A high concentration of acetic acid is also present in lignocellulosic hydrolysates, and it is desirable to develop tolerant yeast for the efficient bioconversion of lignocellulosic biomass [18]. An overdose of acetic acid is toxic to microbial growth and metabolism, and thereby this organic acid is also used in food preservation [19]. In *S. cerevisiae*, acetic acid dissociates into protons and acetate anions under intracellular neutral conditions, causing a reduction in intracellular pH and the inhibition of metabolic activity. The excessive accumulation of ions will lead to an enhancement in ROS levels, resulting in oxidative stress and even acetic acid-induced regulated cell death (AA-RCD) [20,21]. Through the influence on cell apoptosis, acetic acid can accelerate aging and shorten the CLS of yeast [22]. Hence, yeast acetic acid stress also serves as a model for human aging and longevity [23]. Therefore, exploring the mechanisms underlying yeast acetic acid stress tolerance is of great significance for yeast biotechnology applications, human life span, and disease treatments.

Some studies have already reported key genes and metabolic pathways related to acetic acid stress tolerance, including the gluconeogenesis pathway, de novo purine metabolism, acetate transport, and histone modification [23,24,25,26,27]. Protein kinases of some stress signaling pathways which are conserved in eukaryotes have been shown to be involved in the response to acetic acid stress and help cells to adapt to the stress. These include MAPK (mitogen-activated protein kinase), RTG (retrograde), TOR (target of rapamycin), and Ras-cAMP-PKA (Ras-cyclic AMP-dependent protein kinase A), which have been reported to contribute to the acetic acid stress response and the regulation of AA-RCD [28,29]. To uncover unidentified mechanisms underlying stress tolerance in yeast, systems biology approaches, such as transcriptomics, proteomics, and metabolomics, are commonly applied [30]. In our previous study, proteomic studies revealed that protein kinases Kic1 and Hog1 contribute to acetic acid stress tolerance in *S. cerevisiae*, and the protein kinase Rim15 was also identified to contribute to acetic acid stress tolerance [31,32]. Rim15 has a higher abundance in flocculating yeast than in its non-flocculating mutant under acetic acid stress, and the regulation of flocculation by Rim15 was also reported [32,33]. Recent studies have shown that Rim15 is also involved in responses to heavy metals, heat shock, and saline stress [34,35]. Although Rim15 is involved in responses to multiple stresses, which are related to oxidative stress induction, the precise mechanism of Rim15 in stress response and redox biology remains unclear. In addition, although transcriptomic analyses are widely applied in studies on stress tolerance, so far limited studies on transcriptional regulation by Rim15 under stress conditions have been reported [36].

Here, using acetic acid and peroxide as two example stress factors to induce oxidative stress, we explored how Rim15 regulates stress tolerance and redox biology in the eukaryotic model of budding yeast. This study was performed in three aspects: (1) changes in the activities of antioxidant systems mediated by Rim15 under stress conditions; (2) the transcriptional changes caused by Rim15 under stress conditions; and (3) the roles of Rim15 kinase activity on oxidative stress tolerance and the transcription of target genes. This work uncovers that Rim15 plays important roles in modulating the antioxidant system at various levels, and the results provide new insights into the diverse mechanisms of antioxidant stress responses mediated by Rim15.

## 2. Materials and Methods

### 2.1. Plasmids, Strains, and Culture Media

The plasmids and strains employed in this study are listed in Table S1 and Table S2, respectively. *Escherichia coli* DH5α was used as a host strain to construct all plasmids and was cultured in Luria–Bertani (LB) medium (5 g/L yeast extract, 10 g/L tryptone, and 10 g/L NaCl). The antibiotics ampicillin (100 μg/mL) or kanamycin (100 μg/mL) were added into LB medium to select *E. coli* transformants. *S. cerevisiae* BY4741 was chosen as the parent strain which was cultivated in yeast extract–peptone–dextrose (YPD) medium (10 g/L yeast extract, 20 g/L peptone, and 20 g/L glucose). The YPD medium with the addition of antibiotics geneticin (250 μg/mL), hygromycin B (300 μg/mL), and zhongshengmycin (250 μg/mL, as an alternative to nourseothricin) was used for selection of yeast transformants.

*S. cerevisiae* AH109, which was applied in yeast two-hybrid assays, was cultured in a synthetic dextrose (SD) medium (6.7 g/L yeast nitrogen base without amino acids and with ammonium sulfate, 20 g/L glucose, and 0.6 g/L DO supplement -His/-Leu/-Trp/-Ura). Uracil (20 mg/L), histidine (20 mg/L), tryptophan (40 mg/L), and leucine (60 mg/L) were supplemented into SD medium if necessary.

### 2.2. Plasmid and Strain Construction

Primers for plasmid and strain construction are listed in Table S3. All plasmids were constructed using seamless cloning through ClonExpress^®^ Ultra One Step Cloning Kit (Vazyme, Nanjing, China). Single-gene knock-out strains *rgs2*Δ, *rim15*Δ, *sip18*Δ, *srx1*Δ, and *ydj1*Δ were gifted by Prof. Shanshan Li at Hubei University, and each deletion gene was replaced by a *KanMX* expression cassette. Other yeast strains, derived from *S. cerevisiae* BY4741, were constructed using CRISPR/Cas9-based gene editing technology. Gene overexpression was achieved by replacing the native promoter of *RIM15* with a strong promoter, *TEF1_p_*. The whole-gene or fragment deletion was accomplished by substituting donor DNA containing 500 bp upstream and 500 bp downstream homologous arms of the target sequence. Site-directed mutagenesis was acquired with the PCR-based method using the mismatch primers. The donor DNA was amplified with PCR by using the *S. cerevisiae* BY4741 genome as a template. pRS42H-gRNA plasmid containing specific gRNA sequences for target genes and donor DNA were co-transformed into a strain containing p414-Cas9 or Cas9-NAT plasmid [37]. Cas9-NAT plasmid was gifted by Prof. Yueqin Tang at Sichuan University [38]. The yeast transformants were selected from YPD agar plates with corresponding antibiotics, which were verified with PCR and sequencing, and cured in fresh YPD medium without selective antibiotics.

### 2.3. Estimation of Yeast Growth

Stress tolerance of yeast strains was evaluated with both spot assay and growth monitoring during liquid culture and fermentation. For the spot assay, after culturing in a 5 mL centrifuge tube with 1 mL YPD liquid medium at 30 °C and 150 rpm for 24 h, the yeast cells were transferred to a 50 mL centrifuge tube with 5 mL YPD agar medium and cultured at 30 °C and 150 rpm. After 12 h of incubation, the log-phase cells were harvested and adjusted to OD_600_ of 1.0 with sterile water. Two microliters of 10-fold serial diluted suspensions were spotted on YPD agar plates with 4.2 g/L acetic acid or without inhibitors. The plates were then incubated at 30 °C for 36–48 h.

Liquid culture was performed in 250 mL flasks with 100 mL YPD fermentation medium (4 g/L yeast extract, 3 g/L peptone, and 100 g/L glucose). Yeast cells were cultured in 50 mL centrifuge tubes containing 10 mL YPD medium at 30 °C, shaking at 150 rpm for 24 h. Then, 1 mL yeast broth was transferred into 100 mL YPD fermentation medium in 250 mL flasks and cultivated at 30 °C and 150 rpm for 12 h. The log-phase cells were collected and inoculated into YPD fermentation medium by adjusting the initial OD_600_ to 0.1 and were cultured at 30 °C and 150 rpm without pH adjustment and culturing for 96 h or until glucose was depleted. Inhibitors, including acetic acid (4.2 or 5 g/L) or H_2_O_2_ (5 or 10 mM), were added into YPD fermentation medium to evaluate fermentation under stress. Samples were taken at 12 h intervals for 96 h or until glucose was depleted. Cell growth was monitored based on the measurement of OD_600_ via a microplate spectrophotometer (MULTISKAN GO, Thermo, Waltham, MA, USA). The concentrations of metabolites, such as glucose and acetic acid, were determined using high-performance liquid chromatography (HPLC, Waters Alliance e2695 HPLC, Waters, Milford, MA, USA) with a carbohydrate analysis column (Aminex^®^ HPX-87H column, Bio-Rad, Hercules, CA, USA) [39].

### 2.4. Antioxidant Indicator Measurement

For ROS accumulation measurement, yeast cells at the log phase of fermentation were harvested and washed with PBS buffer twice. ROS accumulation was measured using a Reactive Oxygen Species Assay Kit (S0033S, Beyotime, Shanghai, China) based on oxidant-sensitive probe 2′,7′-dichlorofluorescin diacetate (DCFH-DA) following the manufacturer’s instructions. Non-fluorescent DCFH-DA can be oxidized by ROS to the fluorescent compound 2’,7’-dichlorofluorescein (DCF), and the fluorescence intensity was detected at the excitation wavelength of 488 nm and the emission wavelength of 525 nm using a multimode microplate reader (Spark, Tecan, Männedorf, Switzerland). To eliminate variation between different batches of samples, ROS fold change was calculated by dividing the fluorescence value of the experimental group by the fluorescence value of the negative control group without the addition of the fluorescent probe.

For the measurement of other antioxidant parameters, including total antioxidant capacity, ROS, CAT, SOD, and GPx activities, GSH and oxidized glutathione disulfide (GSSG), and glutathione reductase (GR) activity, log-phase yeast cells were collected and lysed with glass beads. The abundance of total cell protein from lysates was measured using a BCA Protein Quantification Kit (Vazyme, Nanjing, China) for normalized calculation. Total antioxidant capacity was measured using a Total Antioxidant Capacity Assay Kit with a Rapid ABTS method (S0121, Beyotime, Shanghai, China) following the manufacturer’s instructions. Total antioxidant capacity was calculated through colorimetric analysis for green ABTS^·+^ product at A_414_ using a microplate spectrophotometer (MULTISKAN GO, Thermo, Waltham, MA, USA).

CAT activity was measured using a Catalase Assay Kit (S0051, Beyotime, Shanghai, China) based on the manufacturer’s instructions. The CAT activity was calculated through colorimetric analysis for a red product at A_520_. SOD activity was measured using a Total Superoxide Dismutase Assay Kit with WST-8 (S0101S, Beyotime, Shanghai, China) following the manufacturer’s instructions. The SOD activity was calculated through colorimetric analysis for formazan dye product at A_450_. GPx activity was measured using a Cellular Glutathione Peroxidase Assay Kit with DTNB (S0057S, Beyotime, Shanghai, China) following the manufacturer’s instructions. GSH and GSSG contents were measured using the GSH and GSSG Assay Kit (S0053, Beyotime, Shanghai, China) based on the DTNB method. The sample was divided into two parts: one for converting GSSG to GSH to determine the total contents of GSSG and GSH, and the other one was used to remove GSH and then convert GSSG into GSH to determine the content of GSSG. The contents of GSH and GSSG and the ratio of GSH/GSSG were then calculated. GR activity was measured using a Glutathione Reductase Assay Kit with DTNB (S0055, Beyotime, Shanghai, China) following the instructions from the manufacturer. GPx activity, GSH content, and GR activity were all calculated through colorimetric analysis for yellow TNB product at A_412_.

### 2.5. Transcriptome Analysis

The log-phase yeast cells were harvested from the fermentation broth, washed with sterile water, and frozen with liquid nitrogen for transcriptome determination. RNA sequencing and transcriptome analysis were carried out by Beijing Novogene Technology Co., Ltd., Beijing, China.

### 2.6. Real-Time Quantitative PCR Analysis

The yeast cells at the log phase of fermentation were collected for RNA extraction. Total RNA was isolated using Hipure Yeast/Bacterial RNA Kit (Magen, Guangzhou, China); then, 1 μg RNA was reversely transcribed via Goldensta^TM^ RT6 cDNA Synthesis Kit Ver.2 (Tsingke, Beijing, China) to obtain cDNA. Real-time quantitative PCR (RT-qPCR) was performed with 2×TSINGKE^®^ Master qPCR Mix (SYBR Green I) (Tsingke, Beijing, China) using Real-Time PCR Detection Systems (CFX Connect^TM^, Bio-Rad, Hercules, CA, USA). All experiments were carried out according to the corresponding manufacturer’s instructions. The relative expression levels of genes were calculated with 2^−ΔΔCT^ and normalized using the values of the reference gene *ALG9* [40].

### 2.7. Yeast Two-Hybrid Assays

Yeast strain AH109 was employed for yeast two-hybrid assays to verify the specific protein–protein interactions. Target genes were amplified from BY4741 genome using PCR and then fused to plasmids pGADT7 and pGBKT7 containing transcription-activation domain (AD) and DNA-binding domain (BD), respectively. Plasmids derived from pGADT7 (pGADT7-Yap1) and pGBKT7 (pGBKT7-Rim15^1-344^ and pGBKT7-Rim15^359-1771^) were co-transformed into AH109 as the experimental groups. Gal4^AD^ and Gal4^BD^ plasmids were transformed individually or co-transformed with the other empty vector as control groups to exclude the possibility of self-activation. The verification of self-activation was performed using SD agar plates with a lack of different amino acids (SD -Leu/-His/-Ade, SD -Trp/-His/-Ade, and SD -Leu/-Trp/-His/-Ade). The yeast two-hybrid assays, according to the principle of activation of report genes *ADE2* and *HIS3*, were carried out on SD -Leu/-Trp/-His, SD -Leu/-Trp/-Ade, or SD -Leu/-Trp/-His/-Ade agar plates. Furthermore, the assays based on β-galactosidase activity were conducted on the SD Leu/-Trp/-His/-Ade agar plates with *X-gal* overlay. The experimental method of spotting is described above. The plates were incubated at 30 °C for 60 to 72 h.

### 2.8. Statistical Analysis

Statistical analysis was performed using Prism 9.0 statistical analysis software (GraphPad Software, Boston, MA, USA). All experiments were repeated at least three times. The data were analyzed with a *t* test, and the significant levels were indicated as follows: * *p* < 0.05, ** *p* < 0.01, and *** *p* < 0.001.

## 3. Results

### 3.1. Rim15 Regulates Yeast Acetic Acid Stress Tolerance

**Figure 1:**
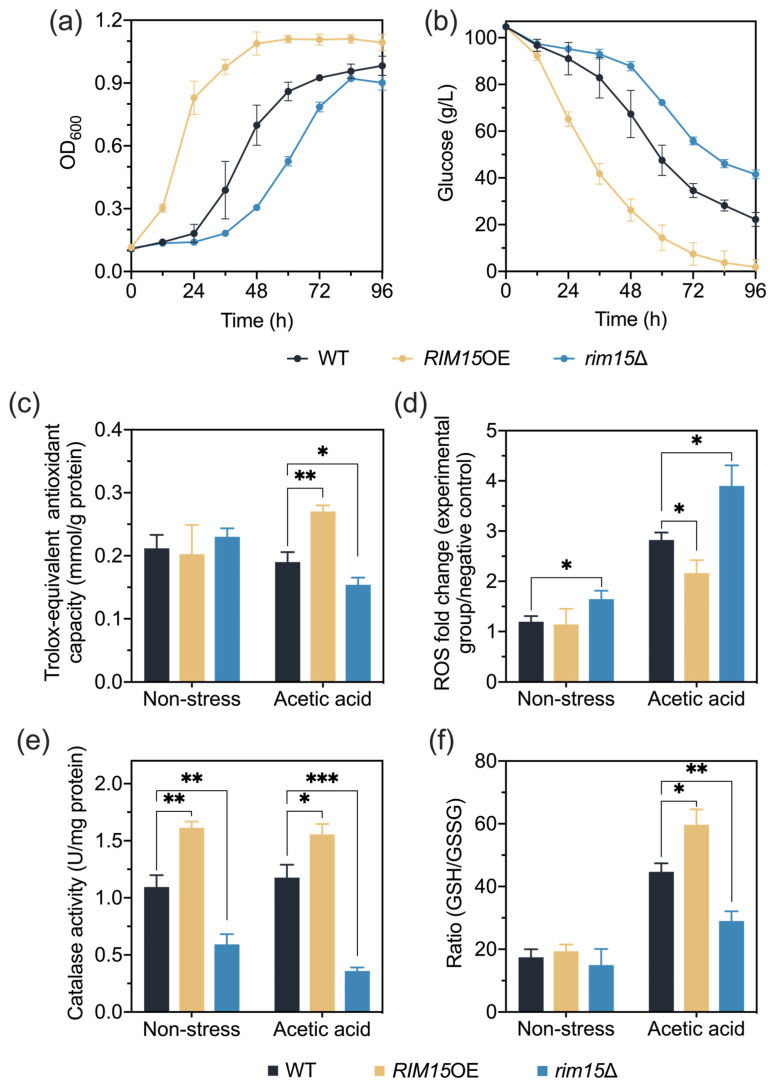
*RIM15* influenced acetic acid stress tolerance by mediating antioxidant systems. (**a**,**b**) Fermentation was performed in 250 mL flasks with YPD fermentation medium supplemented with 4.2 g/L acetic acid using *RIM15*OE, *rim15*Δ, and BY4741 strains. Total antioxidant capacity (**c**), ROS accumulation (**d**), CAT activity (**e**), and ratio of GSH to GSSG (**f**) of *RIM15*OE, *rim15*Δ, and BY4741 strains were measured with or without the treatment with 4.2 g/L acetic acid. Biological triplicates were employed. Error bars represent the standard deviations. Statistical analysis was performed with a *t* test, and the significant levels are indicated as follows: * *p* < 0.05, ** *p* < 0.01, *** *p* < 0.001.

Our previous studies have revealed that the overexpression of gene *RIM15* enhanced acetic acid tolerance in the industrial yeast strain *S. cerevisiae* PLY01 [32]. To comprehensively investigate the biological function of Rim15, phenotypical analysis was performed using *RIM15* overexpression and deletion strains derived from the *S. cerevisiae* laboratory strain BY4741. Since the stress tolerance of the laboratory strain seems to be weaker than that of industrial yeast, lower acetic acid concentrations of 4.2 g/L (compared to 5.0 g/L acetic acid for industrial yeast in the previous study) were chosen for the tolerance test in this study [32]. Under stress-free conditions, the overexpression or deletion of *RIM15* did not affect yeast growth (Figure S1a,b). In contrast, under acetic acid stress, *RIM15*OE showed better growth and faster glucose consumption than the wild-type strain. On the other hand, yeast cells lacking *RIM15* were hypersensitive to acetic acid and were even unable to survive with the treatment with 5 g/L acetic acid (Figure 1a,b and Figure S1c,d). These results further demonstrated that Rim15 plays a critical role in yeast acetic acid stress tolerance.

To test whether the effect of Rim15 is related to acetic acid degradation, we detected the concentration of acetic acid in the medium. No changes throughout the culture of all the strains were found (Figure S2a,b), indicating that acetic acid was not consumed and converted during growth. The toxicity of acetic acid is mainly evoked by cell acidification and increased intracellular ROS levels causing oxidative stress [20]. To further explore the effect of Rim15 on cells under acetic acid stress conditions, a tolerance evaluation was performed at pH 3.5, which is equal to the pH of medium containing 5 g/L acetic acid. The same pH can simulate a similar acidic environment and cell acidification to that under acetic acid stress. Of note, strains BY4741, *RIM15*OE, and *rim15*Δ exhibited a similar growth profile when cultivated at pH 3.5, indicating that the effect of *RIM15* overexpression and deletion under acetic acid stress is not due to the mechanisms related to low pH (Figure S2c).

### 3.2. Rim15 Regulates Acetic Acid Stress Tolerance through Mediating Antioxidant Systems

Total antioxidant capacity can reflect the ability to remove the excessive accumulation of intracellular ROS [41]. To further explore whether Rim15 improved acetic acid stress tolerance through alleviating oxidative stress, total antioxidant capacity and interior ROS levels of strains BY4741, *RIM15*OE, and *rim15*Δ were measured under non-stress and acetic acid stress (4.2 g/L) conditions. Consistent with the growth performance (Figure 1a,b and Figure S1a,b), there was no significant difference in the total antioxidant capacity of all strains incubated without inhibitors. In contrast, under acetic acid stress conditions, we found that the total antioxidant capacity of *RIM15*OE was improved, whereas that of *rim15*Δ was reduced (Figure 1c). In agreement with the antioxidant capacity, under acetic acid stress conditions, the ROS levels of *RIM15*OE and *rim15*Δ were found to be higher and lower than that of the wild-type BY4741, respectively (Figure 1d). These results suggest the important role of Rim15 in improving total antioxidant capacity for scavenging intracellular ROS.

The excessive accumulation of ROS could be scavenged by antioxidant enzymes, including SOD, CAT, and GPx [13]. Thus, antioxidant enzyme activities were examined. Under both non-stress and acetic acid stress conditions, *RIM15*OE contained higher CAT activities than BY4741, while *rim15*Δ contained lower CAT activities than BY4741 (Figure 1e). These results indicated that overexpression of *RIM15* could constitutionally enhance CAT activities, contributing to the rapid removal of ROS under acetic acid stress without induction. However, the SOD activities of yeast strains BY4741, *RIM15*OE, and *rim15*Δ under all conditions were at the same levels (Figure S3a). The GPx activities could not be detected in our experiments; GPx might not be the key enzyme affected by Rim15 for combating oxidative stress.

In addition to the enzymatic antioxidants, the small antioxidant molecule GSH is also involved in scavenging intracellular ROS [15]. Thus, the contents of GSH and GSSG were measured (Figure S3b,c), and the ratio of GSH/GSSG was calculated with or without the treatment with 4.2 g/L acetic acid. The ratio of GSH/GSSG was increased in *RIM15*OE and decreased in *rim15*Δ under acetic acid stress conditions (Figure 1f). GR is known to mediate the conversion from GSSG to GSH [42]. The activities of GR under non-stress and acetic acid stress conditions could explain the changes in the ratio of GSH/GSSG (Figure S3d). These results suggest that, under acetic acid stress conditions, Rim15 regulates the ratio of GSH/GSSG through the GR activity to remove ROS. Altogether, it appears that Rim15 improves the total antioxidant capacity of yeast cells, especially through modulating CAT activity and the GSH/GSSG ratio, leading to enhanced acetic acid stress tolerance.

### 3.3. Transcriptomic Analysis Reveals Global Effects of Rim15 in Transcription

**Figure 2:**
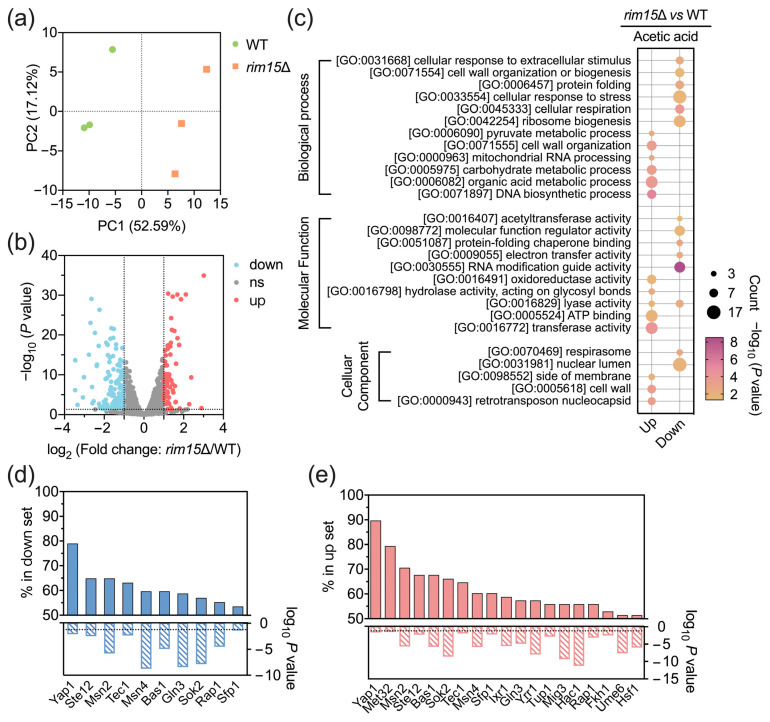
Rim15 regulated acetic acid stress tolerance at the transcriptional level. (**a**) Principal component analysis of transcriptomic data with 3 replicates each in *rim15*Δ and BY4741 strains. The first principal component and second principal component are shown on the *X*-axis and *Y*-axis, respectively. (**b**) Volcano plots reveal the differentially expressed genes from transcriptomics. The vertical dashed lines represent log_2_ (fold change) of ±1. The horizontal dashed line represents a *p* value of 0.05. ns, no significance. (**c**) Gene ontology analysis based on enriched DEGs including different pathways of biological processes, molecular functions, and cellular components. (**d**,**e**) The transcriptional factors regulating DEGs were predicted using the YEASTRACT database (http://www.yeastract.com, accessed on 28 January 2022). The horizontal dashed line represents a *p* value of 0.05.

The comparative transcriptome analysis between *rim15*Δ and BY4741 was performed to display the effects of Rim15 on global gene expression levels under acetic acid stress conditions. Principal component analysis (PCA) of transcriptomic data revealed that *RIM15* obviously influenced the transcription state in yeast grown under acetic acid stress conditions (Figure 2a). There are 201 differentially expressed genes (DEGs), including 69 upregulated genes and 132 downregulated genes (|log_2_ Fold change| > 1, Figure 2b). To better understand the functions of DEGs regulated by Rim15 on acetic acid stress tolerance, gene ontology (GO) analysis was conducted, and the results demonstrated that the downregulated genes were primarily associated with some critical biological processes (Figure 2c), of which cell wall organization or biogenesis was essential for tolerance since the function of the cell wall could be destroyed by acetic acid [43], and the correct protein folding was also important to the regular metabolic activities [44]. The reduced expression levels of genes related to the cell respiration process were apparent with the deletion of *RIM15*.

In previous studies, Rim15 was reported to regulate the stress-responsive genes by interacting with transcriptional factors Gis1, Hsf1, Msn2, and Msn4 in response to nutrient restriction [7,8]. Therefore, the transcriptional factors regulating DEGs under acetic acid stress conditions were predicted. Remarkably, Yap1, a well-known oxidative stress-responsive transcriptional factor, was found to be involved in regulating the expression of approximately 90% of upregulated and 80% of downregulated DEGs (Figure 2d,e). These results indicate the important function of Yap1 in the Rim15-mediated regulation of yeast acetic acid stress response.

### 3.4. RGS2, SIP18, SRX1, and YDJ1 Are Novel Target Genes of Rim15 Which Affect Acetic Acid Tolerance in Yeast

**Figure 3:**
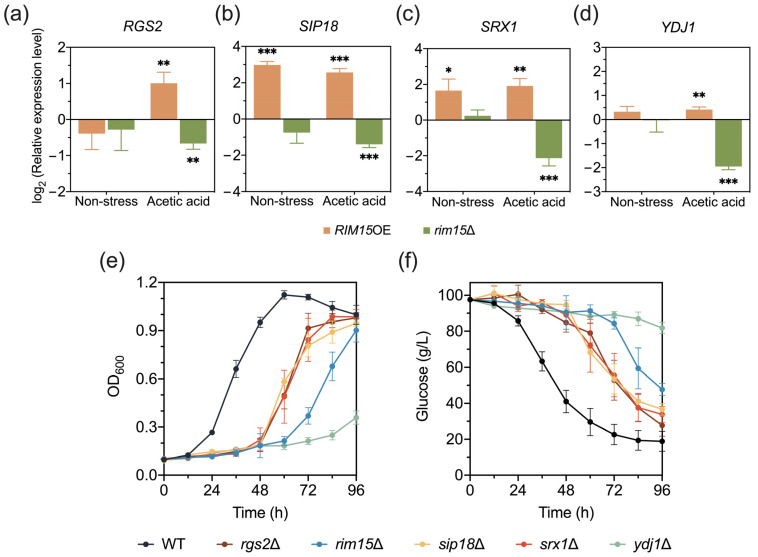
Mining Rim15 target genes related to acetic acid stress tolerance. The changes in the expression levels of target genes *RGS2* (**a**), *SIP18* (**b**), *SRX1* (**c**), and *YDJ1* (**d**) in *RIM15*OE and *rim15*Δ strains were validated with RT-qPCR under non-stress and 4.2 g/L acetic acid stress conditions, respectively. (**e**,**f**) Evaluation of fermentation performance with BY4741 and gene knock-out strains *rgs2*Δ, *rim15*Δ, *sip18*Δ, *srx1*Δ, and *ydj1*Δ was performed after the treatment with 4.2 g/L acetic acid. Biological triplicates were employed. Error bars represent the standard deviations. Statistical analysis was performed with a *t* test, and the significant levels are indicated as follows: * *p* < 0.05, ** *p* < 0.01, *** *p* < 0.001.

Since Rim15 can regulate stress-responsive genes, the specific target genes under acetic acid stress conditions were further explored. Firstly, 18 genes were chosen from the DEGs due to their important functions in cellular activities and potential relation to the stress response (Table S4). Afterward, the transcription levels of the selected genes in the yeast strains were analyzed with RT-qPCR under acetic acid stress conditions, and eight out of eighteen genes were further focused on based on their opposite changes in the expression levels in *RIM15*OE and *rim15*Δ compared with those of the control strains (Figure 3a–d). To verify the functions of the eight potential target genes, a spot assay and growth under acetic acid stress and non-stress conditions using knock-out strains were performed. Notably, among the eight deletion strains tested, *rgs2*Δ, *sip18*Δ, *srx1*Δ, and *ydj1*Δ exhibited reduced acetic acid stress tolerance compared with BY4741 (Figure 3e,f and Figure S4). Except for Ydj1 (type I HSP40 co-chaperone) [45], there is no report showing the involvement of Rgs2 (regulator of G-protein signaling protein), Sip18 (phospholipid-binding hydrophilin), and Srx1 (sulfiredoxin) in yeast acetic acid stress tolerance. Therefore, *RGS2*, *SIP18*, *SRX1*, and *YDJ1* were regarded as the novel target genes regulated by Rim15 under acetic acid stress conditions.

### 3.5. Rim15 Functions with Yap1 in Transcriptional Regulation under Acetic Acid Stress

**Figure 4:**
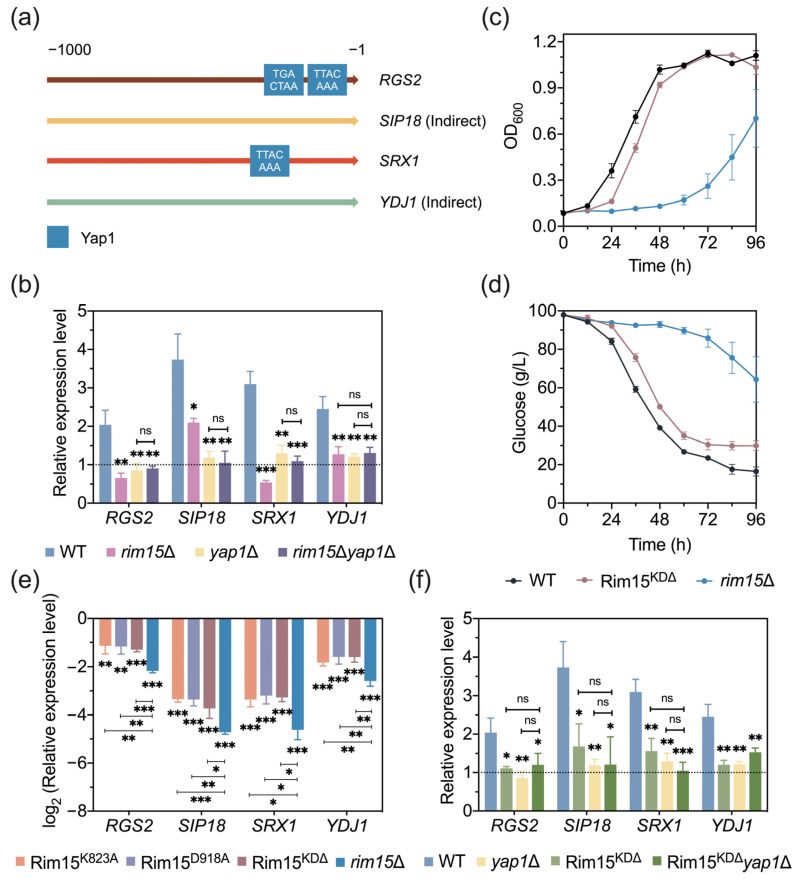
Rim15 exerts transcriptional regulation through Yap1 to improve acetic acid stress tolerance, which is related to its kinase activity. (**a**) Yap1-binding sites in the promoter region of *RGS2*, *SIP18*, *SRX1*, and *YDJ1* were predicted using the YEASTRACT database (http://www.yeastract.com). (**b**) The differences in the expression levels of target genes among BY4741, *rim15*Δ, *yap1*Δ, and *rim15*Δ*yap1*Δ strains were measured with RT-qPCR with or without the treatment with 4.2 g/L acetic acid. (**c**,**d**) Fermentation of BY4741, Rim15^KDΔ^, and *rim15*Δ strains was evaluated under 4.2 g/L acetic acid. (**e**) Transcription of target genes *RGS2*, *SIP18*, *SRX1*, and *YDJ1* in Rim15 kinase-dead strains (Rim15^K823A^, Rim15^D918A^, and Rim15^KDΔ^) and *rim15*Δ was detected with RT-qPCR with the treatment with 4.2 g/L acetic acid. (**f**) The differences in the expression levels of target genes among BY4741, Rim15^KDΔ^, *yap1*Δ, and Rim15^KDΔ^*yap1*Δ strains were measured with RT-qPCR under non-stress and 4.2 g/L acetic acid stress conditions. ns, no significance. Biological triplicates were employed. Error bars represent the standard deviations. Statistical analysis was performed with a *t* test, and the significant levels are indicated as follows: * *p* < 0.05, ** *p* < 0.01, *** *p* < 0.001.

According to previous studies, Rim15 functions in regulating gene expression levels through the regulation of specific transcriptional factors [7,8]. Therefore, the transcriptional factors regulating *RGS2*, *SIP18*, *SRX1*, and *YDJ1* were predicted using YEASTRACT. Yap1-binding sites were present in the promoter regions of *RGS2* and *SRX1* among the four genes (Figure 4a), suggesting the direct regulation of these two genes and the indirect regulation of *SIP18* and *YDJ1*. To confirm the function of Yap1 in regulating the target genes of Rim15, the expression levels of *RGS2*, *SIP18*, *SRX1*, and *YDJ1* were tested in BY4741 and *yap1*Δ under non-stress and acetic acid stress conditions. Upon the treatment with 4.2 g/L acetic acid, the expression of these four genes was induced in BY4741. Moreover, their expression levels were not so significantly induced in the *YAP1* deletion strain, and Rim15 deletion in *yap1*Δ did not further inhibit the induction, indicating that Rim15 functions together with Yap1 in regulating acetic acid stress-induced gene expression (Figure 4b).

Rim15 kinase-dead strains Rim15^K823A^ (ATP-binding site mutant), Rim15^D918A^ (proton acceptor mutant), and Rim15^KDΔ^ (kinase domain mutant with a deletion of Rim15 794–1254 amino acids) were constructed, and the effect of Rim15 kinase activity on yeast growth was evaluated. There are no differences among the growth and glucose consumption of BY4741, Rim15 kinase-dead strains, and *rim15*Δ under non-stress conditions (Figure S5a,b). In contrast, under acetic acid stress conditions, the stress tolerances of all Rim15 kinase-dead strains were lower than those of BY4741, but their growth was much better than that of *rim15*Δ (Figure 4c,d and Figure S5c,d). These results suggest that although the kinase function of Rim15 is necessary for the regulation of acetic acid stress tolerance, some other activities of Rim15, besides kinase activity, seem to be more critical for conferring acetic acid stress tolerance. In addition, Rim15 kinase activity was found to be involved in the transcriptional regulation of acetic acid stress-related genes (Figure 4e). To explore the relationship between kinase Rim15 and transcriptional factor Yap1, the expression levels of *RGS2*, *SIP18*, *SRX1*, and *YDJ1* were tested in *yap1*Δ and Rim15^KDΔ^*yap1*Δ under acetic acid and non-stress conditions. The expression levels of these target genes were not further decreased in Rim15^KDΔ^*yap1*Δ compared with *yap1*Δ and Rim15^KDΔ^ strains (Figure 4f), indicating that Rim15 regulates Yap1 through its kinase activity. However, the yeast two-hybrid test showed no direct interaction between Rim15 and Yap1 (Figure S6), implying that additional protein(s) may be required for Rim15 to regulate Yap1 target genes in response to acetic acid.

Intriguingly, compared to BY4741, the expressional levels of target genes *RGS2*, *SRX1*, and *YDJ1* in all mutants did not change under non-stress conditions but reduced after the treatment with 4.2 g/L acetic acid, only *SIP18* was constitutionally suppressed even without inhibitors, and the expression levels were further decreased by the treatment with acetic acid (Figure 4e and Figure S5e). These results indicate different regulatory mechanisms for controlling the expression of *SIP18* and the three other target genes of Rim15.

### 3.6. Rim15 Improves H_2_O_2_ Stress Tolerance through Antioxidant Systems

**Figure 5:**
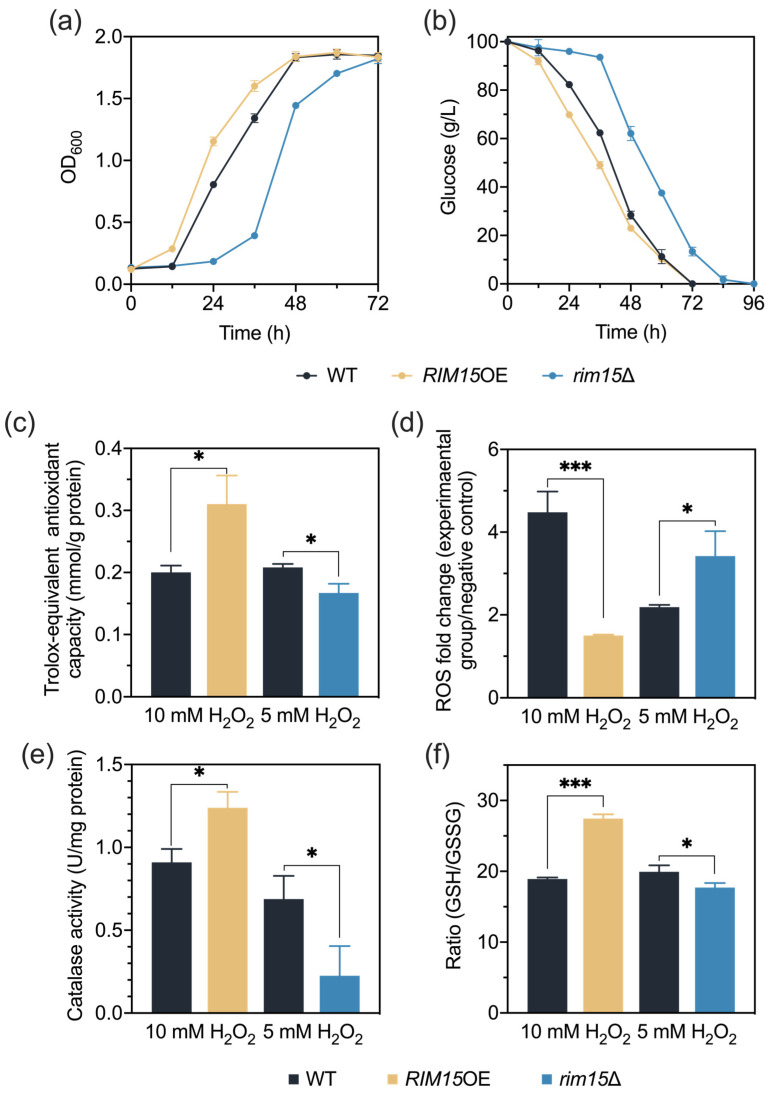
Rim15 regulated the antioxidant systems to improve oxidative stress tolerance. (**a**,**b**) Fermentation was performed in 250 mL flasks with YPD fermentation medium supplemented with 5 mM H_2_O_2_ using *RIM15*OE, *rim15*Δ, and BY4741 strains. Total antioxidant capacity (**c**), ROS accumulation (**d**), CAT activity (**e**), and ratio of GSH to GSSG (**f**) of *RIM15*OE, *rim15*Δ, and BY4741 yeast strains were measured with the treatment with H_2_O_2_. Biological triplicates were employed. Error bars represent the standard deviations. Statistical analysis was performed with a *t* test, and the significant levels are indicated as follows: * *p* < 0.05, *** *p* < 0.001.

Oxidative stress is one of the serious consequences caused by acetic acid stress [20]. To further test whether Rim15 contributed to the resistance to oxidative stress, the growth of strains BY4741, *RIM15*OE, and *rim15*Δ was investigated using H_2_O_2_ as a stress elicitor. Similar to the performance under acetic acid stress conditions, the overexpression of *RIM15* led to an enhanced tolerance, and *rim15*Δ showed severe growth and fermentation defects when treated with 5 mM H_2_O_2_ (Figure 5a,b), which is consistent with a previous report [6]. When the H_2_O_2_ concentration was increased to 10 mM, *rim15*Δ could not survive (Figure S7). The quantification of antioxidant indicators of strains BY4741, *RIM15*OE, and *rim15*Δ under H_2_O_2_ stress (Figure 5c–f and Figure S8) exhibited similar results to that examined under acetic acid stress conditions (Figure 1c–f and Figure S3). These results demonstrated that Rim15 can also regulate the tolerance to another type of oxidative stress, which is triggered by H_2_O_2_, not acetic acid.

### 3.7. Rim15 Improves H_2_O_2_ Stress Tolerance through Transcriptional Regulation

**Figure 6:**
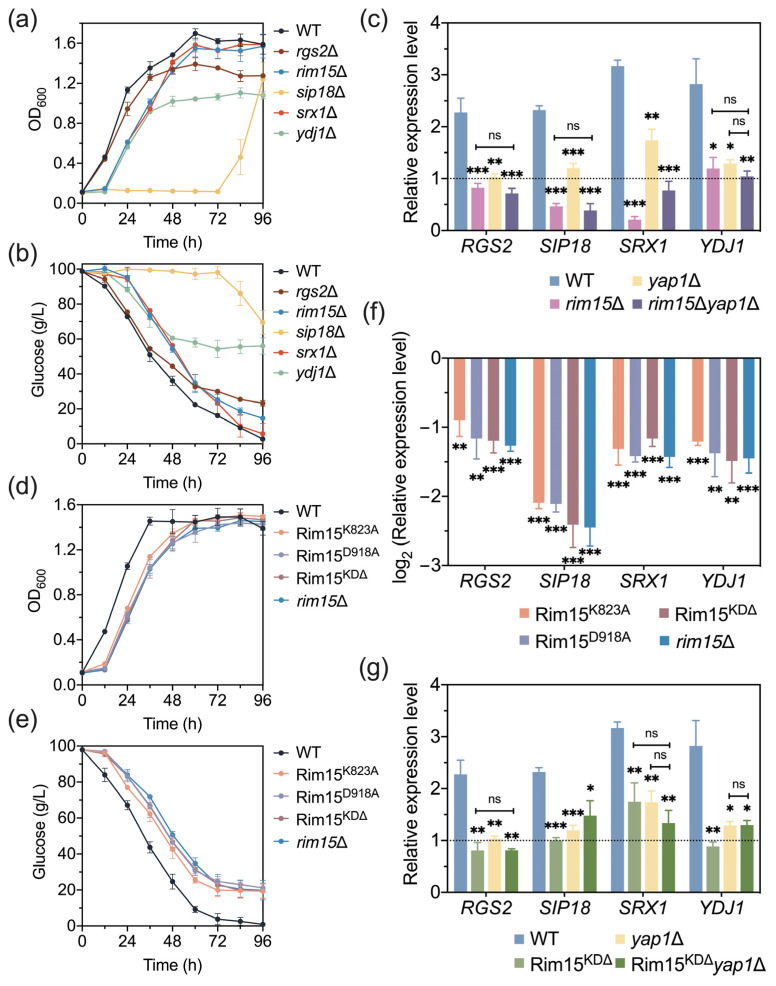
Rim15 regulated stress-induced genes to alleviate oxidative stress triggered by acetic acid. (**a**,**b**) Fermentation of BY4741 and gene knock-out strains *rgs2*Δ, *rim15*Δ, *sip18*Δ, *srx1*Δ, and *ydj1*Δ was evaluated under 5 mM H_2_O_2_. (**c**) The differences in the expression levels of target genes between BY4741 and *yap1*Δ were validated with RT-qPCR with or without the treatment with 5 mM H_2_O_2_. (**d**,**e**) Evaluation of fermentation performance with BY4741, Rim15 kinase-dead yeast strains (Rim15^K823A^, Rim15^D918A^, and Rim15^KDΔ^), and *rim15*Δ was performed with the treatment with 5 mM H_2_O_2_. (**f**) Transcription of target genes *RGS2*, *SIP18*, *SRX1*, and *YDJ1* in Rim15 kinase-dead strains and *rim15*Δ was detected with RT-qPCR under 5 mM H_2_O_2_. (**g**) The differences in the expression levels of target genes among BY4741, Rim15^KDΔ^, *yap1*Δ, and Rim15^KDΔ^*yap1*Δ strains were validated with RT-qPCR with or without the treatment with 5 mM H_2_O_2_. ns, no significance. Biological triplicates were employed. Error bars represent the standard deviations. Statistical analysis was performed with a *t* test, and the significant levels are indicated as follows: * *p* < 0.05, ** *p* < 0.01, *** *p* < 0.001.

Although the relationship between acetic acid stress tolerance and some target genes remains unclear, genes *RGS2*, *SIP18*, *SRX1*, and *YDJ1* were proved to contribute to oxidative stress tolerance [46,47,48,49]. To explore the roles of Rim15 target genes in relieving oxidative stress, the growth performance of the deletion strains lacking Rim15 target genes was evaluated with the treatment of 5 mM H_2_O_2_. Consistent with the results under acetic acid stress conditions (Figure 3e,f), all mutants (i.e., *rgs2*Δ, *sip18*Δ, *srx1*Δ, and *ydj1*Δ) exhibited a decreased tolerance to H_2_O_2_ (Figure 6a,b). Moreover, under oxidative stress conditions, the expression of the four target genes in *yap1*Δ and *rim15*Δ*yap1*Δ was repressed to a similar degree (Figure 6c). These results suggest that Rim15 affects the expression of target genes through the transcriptional factor Yap1, thereby leading to improved oxidative stress tolerance.

In addition, to analyze the effect of Rim15 kinase function on oxidative stress tolerance regulation, the growth performance of BY4741, Rim15 kinase-dead strains, and *rim15*Δ was also evaluated. Notably, unlike the results under acetic acid stress conditions (Figure 4d,e and Figure S5c,d), no significant differences among the stress tolerance of Rim15^K823A^, Rim15^D918A^, Rim15^KDΔ^, and *rim15*Δ were observed, which exhibited reduced tolerance to H_2_O_2_ when compared with BY4741 (Figure 6d,e). These results suggest that the influences of Rim15 on H_2_O_2_ stress tolerance rely on its kinase function. Moreover, the expression levels of target genes in BY4741, Rim15 kinase-dead strains, and *rim15*Δ upon the treatment with 5 mM H_2_O_2_ further proved this perspective (Figure 6f). These results indicate that the kinase function of Rim15 is involved in alleviating oxidative stress, but some other kinase-independent activities of Rim15 are also required for acetic acid stress tolerance. The results in Figure 6g also reveal the relationship between Yap1 and Rim15 kinase activity in transcriptional regulation under oxidative stress.

Given that the target genes of Rim15 contribute to oxidative stress [46,47,48,49], to investigate if these genes are involved in the Rim15-dependent ROS reduction under various stress conditions, *sip18*Δ and *ydj1*Δ were chosen to determine their ROS levels when incubated under non-stress, 4.2 g/L acetic acid, and 5 mM H_2_O_2_ conditions. The results showed that *sip18*Δ and *ydj1*Δ rapidly accumulated ROS under acetic acid and oxidative stress conditions (Figure S9), suggesting the role of Rim15 in reducing ROS accumulation through the regulation of its target genes.

## 4. Discussion

Rim15 was initially discovered as an integrator of the PKA and TORC1 pathways in response to nutrient depletion [2]. In our previous study, Rim15 was found to be more abundant in the industrial flocculating strain SPSC01 with improved stress tolerance, and the overexpression of Rim15 also enhances the acetic acid tolerance of the industrial yeast strain [32]. Here, the functional mechanisms of Rim15 in oxidative stress tolerance in *S. cerevisiae* were further explored. We present evidence that Rim15 regulates oxidative stress tolerance through antioxidant systems and transcriptional regulation, and the kinase function of Rim15 plays an important role in the regulation. Also, our data indicate that redox regulation by Rim15 differs under different stress conditions (acetic acid and H_2_O_2_).

In this study, the overexpression of *RIM15* conferred enhanced oxidative stress tolerance triggered by acetic acid and H_2_O_2_, and this process appears to rely on the higher antioxidant capacity, especially CAT and GSH, to mitigate excessive ROS (Figure 1 and Figure 5). ROS have been implicated in the initiation and progression of cancer, and antioxidants are used in cancer therapy through scavenging ROS [50]. However, several studies have proposed the perspective that antioxidants will protect both tumor cells and normal cells from oxidative stress induced by cancer therapy, which in turn leads to the reduced survival of patients instead [51]. It is also suggested that the antioxidant supplement during therapy is associated with an increased risk of cancer cell metastasis and the hazard of recurrence [52,53]. Moreover, anticancer therapy based on increasing intratumor ROS levels is being developed [54]. In addition, the overexpression of Mastl, the homolog of Rim15 in mammals, contributes to the tumorigenic processes and is even related to the recurrence after initial treatment [55,56]. Combined with our results, it might be possible that carcinogenesis resulting from the overexpression of Mastl may be related to the enhanced antioxidant content and antioxidant ability in tumor cells. It is thus meaningful to further study the treatment of Mastl-mediated tumorigenesis using proper antioxidant treatments.

After mining in the DEGs of the comparative transcriptome, the expression of the genes *RGS2*, *SIP18*, *SRX1*, and *YDJ1* was found to be promoted by Rim15 under acetic acid stress conditions, and they were considered as potential target genes of Rim15 (Figure 3). In addition, these genes were proved to be related to acetic acid and H_2_O_2_ stress tolerance (Figure 3e,f and Figure 6a,b). However, the relationship between Rim15 and these target genes and how they regulate stress tolerance in yeast were both unknown. The overexpression of *RGS2* lowers the cAMP amounts, leading to the inhibition of the Ras-cAMP-PKA pathway [46]. The over-activation of the PKA pathway will cause ROS generation and apoptosis [57]. Therefore, under acetic acid and H_2_O_2_ stress conditions, the upregulation of *RGS2* expression could repress the PKA pathway and ROS accumulation, contributing to stress tolerance. Moreover, Rim15 is also negatively regulated by the PKA pathway [2], indicating that Rgs2 may help the activation of Rim15 in turn. As a phospholipid-binding hydrophilin, Sip18 is important to maintain water in the cytoplasm. The deletion of *SIP18* leads to the accumulation of ROS under desiccation stress, indicating the important function of Sip18 in the oxidative stress response [47]. *SRX1* is sulfiredoxin required for the oxidative stress response [48]. Under caloric restriction, Gcn2-dependent Srx1 translation is increased through the inhibition of the Ras-cAMP-PKA pathway, further enhancing the antioxidant ability [58]. Rim15 activation is also based on the PKA pathway inhibition, indicating that Rim15 may involve Srx1 translation regulation. In addition to the function of Ydj1 in protein folding and refolding as an Hsp40 co-chaperone under heat shock conditions, Ydj1 has been discovered to be accumulated in stress granules and is important for the recovery of translation following stresses, and it may be the reason for its role in acetic acid and H_2_O_2_ stress tolerance [59,60]. Therefore, mining for novel stress-response-related target genes promotes an in-depth understanding of stress tolerance mechanisms.

Some Rim15 target genes are conserved among eukaryotes. In mammals, a high expressional level of *RGS2* has been discovered in both gastric and lung cancer cells and is related to high malignancy and poor prognosis [61,62]. According to our results, the human homolog Rgs2 may improve the stress resistance of tumor cells, leading to a compromised immune response. Srx1 contributes to disease therapy due to its important function in oxidative stress response. Srx1 has been discovered to protect intestinal epithelial cells and attenuate apoptosis during colitis [63]. Srx1 also protects the cardiomyocyte from injury upon ischemic cardiovascular diseases and the lung against oxidative stress brought on by cigarette smoke exposure [64,65]. DNAJA1, the human homolog of Ydj1, has been proven to promote the formation of Amyloid beta 42 (trigger of Alzheimer’s disease) and tumor metastasis. Hence, studies on Rim15 and its target genes may be beneficial for the studies on human disease therapy and pathogenesis.

The regulation of gene expression is a complicated process. Transcriptional factors play the most important and direct roles in the process. Rim15 has been reported to phosphorylate and interact with transcriptional factors Hsf1, Msn2, and Msn4 to activate them and regulate stress-related gene expression [8]. Here, Yap1 was proved to induce the expression of Rim15 target genes *RGS2*, *SIP18*, *SRX1*, and *YDJ1* (Figure 4a,b and Figure 6c). Yap1 is an essential regulator in response to oxidative stress and is activated by oxidative stress [66]. Through RNA-seq and microarray analysis, some studies have already reported that these target genes are indirectly and positively regulated by Yap1 when various chemicals trigger oxidative stress, such as *tert*-butyl hydroperoxide (*t*-BHP), arsenic, methyl methane sulphonate (MMS), and selenite [67,68,69]. However, the specific mechanism of Yap1 regulation of the expression of Rim15 target genes *RGS2*, *SIP18*, *SRX1*, and *YDJ1* is unknown. Yap1 activation requires the Gpx3-mediated multiple interdomain disulfide bonds promoted by H_2_O_2_ to form its oxidation state [70]. Gpx3 is a glutathione peroxidase involved in converting from GSH to GSSG [71]. The bias to GSH production caused by Rim15 may cause more Gpx3 function in Yap1 activation. In addition, Rim15 kinase activity was found to be involved in Yap1 regulating stress response gene expression (Figure 4f and Figure 6g). It is reported that Yap1 has a higher level of phosphorylation under oxidative stress. Therefore, Rim15 kinase activity may be related to the phosphorylation state of the activated Yap1 [72]. However, there was no direct interaction between Rim15 and Yap1 detected by the yeast two-hybrid assay (Figure S4), implying that additional protein(s) may be required for Rim15 to regulate Yap1 function. The in-depth mechanisms underlying the regulation of Yap1 by Rim15 during oxidative stress need to be explored further.

We found that Rim15 kinase function is involved in transcriptional regulation under acetic acid and H_2_O_2_ stress conditions (Figure 4e and Figure 6f). The acetic acid stress tolerance is only partially affected by the kinase function of Rim15, but H_2_O_2_ stress tolerance regulation strongly depends on it, indicating the different mechanisms between these two kinds of stressors. Under rapamycin treatment or nitrogen starvation, the GFP-tagged kinase-inactive Rim15 is not observed in the nucleus [73]. The inhibition of Rim15 translocation to the nucleus may repress the phosphorylation and activation of downstream transcriptional factors, which is consistent with the decreased transcriptional level of target genes under acetic acid and H_2_O_2_ stress conditions in our results (Figure 4e and Figure 6f). However, the deletion of *SCH9* can recover the nuclear accumulation of GFP-tagged kinase-inactive Rim15, and sch9Δ also provides yeast with the ability to survive under acetic acid [73,74]. Sch9 is the inhibitor of Rim15 kinase activity and is required for the inactivation of the PKA pathway, which has recently been reported to be related to acetic acid stress tolerance [28,75], indicating the function of the PKA pathway in Rim15 regulating acetic acid stress tolerance.

Our previous study revealed the protein–protein interaction between Rim15 and MAPK Hog1 [32]. Hog1 is the key kinase of the high-osmolarity glycerol (HOG) pathway involved in osmostress adaptation. Under acetic acid stress, Hog1 phosphorylates the aquaglyceroporin Fps1 and promotes its ubiquitylation and degradation, avoiding the uptake of acetic acid and increasing tolerance [76]. Also, Hog1 can regulate the expression of CAT coding gene *CTT1* with the help of Msn2 and Msn4, further removing the abundant intracellular ROS [21]. Both pathways contribute to the acetic acid stress response. The relationship between kinases Rim15 and Hog1 from different signaling pathways indicates the potential crosstalk of the TOR and PKA pathways with the MAPK pathway in response to acetic acid stress.

**Figure 7:**
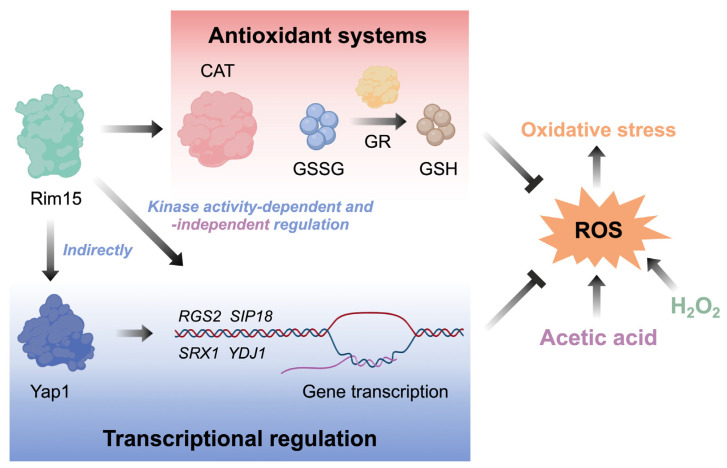
A schematic diagram showing the key events related to Rim15-mediated regulation of stress response. In response to oxidative stress triggered by acetic acid or H_2_O_2_, Rim15 could regulate CAT activities and GSH/GSSH ratio to scavenge abundant ROS and activate the expression of stress-related genes *RGS2*, *SIP18*, *SRX1*, and *YDJ1* through transcriptional factor Yap1. CAT, catalase; GR, glutathione reductase; GSH, reduced glutathione; GSSG, oxidized glutathione disulfide; ROS, reactive oxygen species.

The proposed mechanisms are summarized in Figure 7. Further studies will explore the roles of Rim15 in redox biology in response to various stresses in yeast and other living organisms. Rim15 and some of its target genes are conserved across eukaryotes, and our results thus also imply the important roles of Rim15 in oxidative stress-related processes in various eukaryotic organisms.

## 5. Conclusions

In this study, the involvement of *RIM15* in yeast redox biology was examined using acetic acid and H_2_O_2_ as stress factors. The results revealed that Rim15 regulated the redox balance under oxidative stress through two pathways: (1) Rim15 affected the total antioxidant capacity, especially CAT activity and GSH metabolism, of yeast cells to enable the effective scavenging of the intracellular ROS and (2) Rim15 induced the expression of target genes through the oxidative stress-responsive transcriptional factor Yap1, and *RGS2*, *SIP18*, *SRX1*, and *YDJ1* are potential Rim15 target genes for the regulation of yeast stress responses. Despite the common mechanisms of Rim15-mediated acetic acid and H_2_O_2_ stress responses, Rim15 kinase activity contributes differently to the different stress responses. Our results provide novel insights into Rim15-mediated redox regulation in eukaryotic cells, especially under stress conditions.

## Data Availability

The raw data of the transcriptome are available from the NCBI Sequencing Read Archive (SRA) with the accession number PRJNA1009144.

## Supplementary Materials

The following supporting information can be downloaded at https://www.mdpi.com/article/10.3390/antiox13030260/s1, Supplementary Table S1: Plasmids used in this study; Supplementary Table S2: Strains used in this study; Supplementary Table S3: Primers used in this study; Supplementary Table S4: Function annotation and changes in transcription level of the selected genes affected by *RIM15* deletion; Supplementary Figure S1: Effects of *RIM15* overexpression and deletion on yeast stress tolerance and fermentation performance. Fermentation of the *RIM15* overexpression strain *S. cerevisiae RIM15*OE and the *RIM15* deletion strain *S. cerevisiae rim15*Δ was performed under non-stress (a,b) and 5 g/L acetic acid stress (c,d) conditions, with *S. cerevisiae* BY4741 as a control strain. Biological triplicates were employed. Error bars represent the standard deviations; Supplementary Figure S2: Effects of *RIM15* overexpression and deletion on acetic acid concentration in the fermentation broth and low pH stress tolerance. Acetic acid consumption was detected in the presence of 4.2 g/L (a) and 5 g/L (b) acetic acid. (c) Growth of *S. cerevisiae RIM15*OE, *rim15*Δ, and BY4741 strains was evaluated under pH3.5. Biological triplicates were employed. Error bars represent the standard deviations; Supplementary Figure S3: Effects of *RIM15* overexpression and deletion on the activities of the antioxidant system under acetic acid stress conditions. SOD activity (a), GSH content (b), GSSG content (c), and GR activity (d) of *S. cerevisiae RIM15*OE, *rim15*Δ, and the control strain BY4741 were measured with or without the treatment with 4.2 g/L acetic acid. Biological triplicates were employed. Error bars represent the standard deviations. Statistical analysis was performed with a *t* test, and the significant levels are indicated as follows: * *p* < 0.05, ** *p* < 0.01, *** *p* < 0.001; Supplementary Figure S4: Evaluation of stress tolerance and fermentation performance of the gene deletion yeast strains. (a) Evaluation of stress tolerance with *S. cerevisiae* BY4741 and the knock-out strains of *S. cerevisiae rgs2*Δ, *rim15*Δ, *sip18*Δ, *srx1*Δ, and *ydj1*Δ was performed using spot assays under non-stress and 4.2 g/L acetic acid stress conditions. (b,c) Growth and fermentation performance of the yeast strains under non-stress conditions. Biological triplicates were employed. Error bars represent the standard deviations; Supplementary Figure S5: Effect of Rim15 kinase activity on fermentation and expression of target genes. Fermentation of *S. cerevisiae* BY4741, Rim15 kinase-dead yeast strains, and knock-out strain *rim15*Δ was evaluated under non-stress (a,b) and 4.2 g/L acetic acid stress (c,d) conditions. (e) Transcription of target genes *RGS2*, *SIP18*, *SRX1*, and *YDJ1* in Rim15 kinase-dead strains (Rim15^K823A^, Rim15D^918A^, and Rim15^KDΔ^) and knock-out strain *rim15*Δ was detected with RT-qPCR analysis without inhibitors. Biological triplicates were employed. Error bars represent the standard deviations. Statistical analysis was performed with a *t* test, * *p* < 0.05, ** *p* < 0.01, *** *p* < 0.001; Supplementary Figure S6: Analysis of protein–protein interaction of Rim15 and Yap1. The self-activation of a single protein (a) and protein with empty vector (b) was verified on SD plates without His and Ade and with *X-gal* overlay. If the colony survived on the SD agar plates lacking His and Ade or turned blue with the supplement of *X-gal*, this indicated the existence of self-activation. (c) Yeast two-hybrid assays were employed to verify the interaction between Rim15 and Yap1 in vivo. If the colony survived on the SD agar plates lacking Leu, Trp, His, and Ade or turned blue with the supplement of X-gal, this indicated that there was an interaction between the two proteins; Supplementary Figure S7: Effect of *RIM15* overexpression and deletion on oxidative stress tolerance and fermentation performance. Growth (a) and glucose consumption (b) of *S. cerevisiae* BY4741, *RIM15*OE, and *rim15*Δ strains were observed in the presence of 10 mM H_2_O_2_. Biological triplicates were employed. Error bars represent the standard deviations; Supplementary Figure S8: Effects of *RIM15* overexpression and deletion on antioxidant status of the yeast strains under oxidative stress. SOD activity (a), GSH (b) and GSSG (c) contents, and GR activity (d) of *S. cerevisiae RIM15*OE, *rim15*Δ, and BY4741 strains were measured with or without the treatment with H_2_O_2_. Biological triplicates were employed. Error bars represent the standard deviations. Statistical analysis was performed with a *t* test, and the significant levels are indicated as follows: * *p* < 0.05, ** *p* < 0.01, *** *p* < 0.001; Supplementary Figure S9: Effect of *SIP18* and *YDJ1* gene deletion on ROS accumulation of *S. cerevisiae* BY4741, *sip18*Δ, and *ydj1*Δ was measured under non-stress, 4.2 g/L acetic acid stress, and 5 mM H_2_O_2_ stress conditions, respectively. Biological triplicates were employed in experiments. Error bars represent the standard deviations. Statistical analysis was performed with a *t* test, and the significant levels are indicated as follows: * *p* < 0.05, ** *p* < 0.01, *** *p* < 0.001.
