# Supplementary material for: Novel Roles of the Greatwall Kinase Rim15 in Yeast Oxidative Stress Tolerance through Mediating Antioxidant Systems and Transcriptional Regulation
Source: Antioxidants (Basel). 2024 Feb 21;13(3):260. doi: 10.3390/antiox13030260 (PMC10967648; doi:10.3390/antiox13030260)
Supplement: Supplementary file 1 [file antioxidants-13-00260-s001.zip › antioxidants-2849957-supplementary.pdf]

SUPPLEMENTARY MATERIALS

Table S1. Plasmids used in this study

Plasmids	Description	Source
p414-Cas9	p414, ARS/CEN, *KanMX*, AmpR, *TEF1p*-*Sp*Cas9-*CYC1t*	Lab preservation
Cas9-NAT	ARS/CEN, NrsR, AmpR, *TEF1p*-*Sp*Cas9-*CYC1t*	Gifted from Prof. Yueqin Tang at Sichuan University [38]
pRS42H-gRNA	pRS42H, 2 μm, *hphNT*, AmpR, *SNR52p*-gRNA-*SUP4t*	Lab preservation
pRS42H-gRNA-5'*RIM15*	Plasmid containing gRNA targeted to 5' terminal of gene *RIM15*	This study
pRS42H-gRNA-*yap1*Δ	Plasmid containing gRNA targeted to coding sequence of *YAP1*	This study
pGADT7	pUC, 2 μm, *LEU2*, AmpR, *ADH1p*-*GAL4* activation domain (AD)-*ADH1t*	Clontech
pGBKT7	pUC, 2 μm, *TRP1*, KanR, *ADH1p*-*GAL4* DNA binding domain (BD) *ADH1t*	Clontech
pGADT7-Yap1	*YAP1* was from BY4741 fused to pGADT7	This study
pGBKT7-Rim15$^{1\text{-}344}$	1-1032 bp of *RIM15* was from BY4741 fused to pGBKT7	This study
pGBKT7-Rim15$^{359\text{-}1771}$	1075-5313 bp of *RIM15* was from BY4741 fused to pGBKT7	This study
pRS42H-gRNA-2716*RIM15*	Plasmid containing gRNA targeted to kinase domain of gene *RIM15*	This study

Table S2. Strains used in this study

**Strains**	**Description**	**Source**
Escherichia coli		
DH5α	For plasmid construction and propagation	Invitrogen Ltd
Saccharomyces cerevisiae		
BY4741	*MATa*, *his3Δ1*, *leu2Δ0*, *met15Δ0*, *ura3Δ0*	Lab preservation
BY4741-Cas9	Transform p414-Cas9 plasmid into BY4741	This study
*RIM15*OE	BY4741, *RIM15p*::*TEF1p*	This study
rim15Δ	BY4741, *rim15Δ*::*KanMX*	Open Biosystems (Gifted from Prof. Shanshan Li at Hubei University)
rgs2Δ	BY4741, *rgs2Δ*::*KanMX*	Open Biosystems (Gifted from Prof. Shanshan Li at Hubei University)
sip18Δ	BY4741, *sip18Δ*::*KanMX*	Open Biosystems (Gifted from Prof. Shanshan Li at Hubei University)
srx1Δ	BY4741, *srx1Δ*::*KanMX*	Open Biosystems (Gifted from Prof. Shanshan Li at Hubei University)
ydj1Δ	BY4741, *ydj1Δ*::*KanMX*	Open Biosystems (Gifted from Prof. Shanshan Li at Hubei University)
yap1Δ	BY4741, *yap1Δ*	This study
rim15Δ-Cas9	Transform Cas9-NAT plasmid into *rim15Δ*	This study
rim15Δyap1Δ	BY4741, *rim15Δ*::*KanMX*, *yap1Δ*	This study
AH109	*MATa*, *trp1-901*, *leu2-3*, *112*, *ura3-52*, *his3-200*, *gal4Δ*, *gal80Δ*, *LYS2*::$GAL1_{UAS}$-$GAL1_{TATA}$-*HIS3*, $GAL2_{UAS}$-$GAL2_{TATA}$-*ADE2*, *URA3*::$MEL1_{UAS}$-$MEL1_{TATA}$-*lacZ*	Clontech
pA-Yap1	Transform pGADT7-Yap1 plasmid into AH109	This study
pB-Rim15$^{1\text{-}344}$	Transform pGBKT7-Rim15$^{1\text{-}344}$ plasmid into AH109	This study
pB-Rim15$^{359\text{-}1771}$	Transform pGBKT7-Rim15$^{359\text{-}1771}$ plasmid into AH109	This study

Table S2. (Continued) Strains used in this study

Strains	Description	Source
pA-Mcm2+pB-Mcm10	Positive control	Lab preservation
pA+pB	Negative control	Lab preservation
pA-Yap1+pB	Transform pGADT7-Yap1 and pGBKT7 plasmids into AH109	This study
pA+pB-Rim15$^{1-344}$	Transform pGADT7 and pGBKT7-Rim15$^{1-344}$ plasmids into AH109	This study
pA+pB-Rim15$^{359-1771}$	Transform pGADT7 and pGBKT7-Rim15$^{359-1771}$ plasmids into AH109	This study
pA-Yap1+pB-Rim15$^{1-344}$	Transform pGADT7-Yap1 and pGBKT7-Rim15$^{1-344}$ plasmids into AH109	This study
pA-Yap1+pB-Rim15$^{359-1771}$	Transform pGADT7-Yap1 and pGBKT7-Rim15$^{359-1771}$ plasmids into AH109	This study
Rim15^{K823A}	BY4741, the codon of Rim15 K823 was replaced with GCG	This study
Rim15^{D918A}	BY4741, the codon of Rim15 D918 was replaced with GCC	This study
Rim15$^{KD\Delta}$	BY4741, 2380-3762 bp of *RIM15* was deleted	This study
Rim15$^{KD\Delta}$-Cas9	Transform Cas9-NAT plasmid into Rim15$^{KD\Delta}$	This study
Rim15$^{KD\Delta}$*yap1*Δ	BY4741, 2380-3762 bp of *RIM15* was deleted, *yap1*Δ	This study

Table S3. Primers used in this study*.

Primers	Sequence (5'-3')
gRNA-5'*RIM15*-F	GATCGCCTGCTGTAGAGGTTCTGG
gRNA-5'*RIM15*-R	AAACCCAGAACCTCTACAGCAGGC
M13-R	CAGGAAACAGCTATGACC
Donor-*TEF1p*-5'*RIM15*-F	ATTGTCTGCCGTTTTTTGTATAGATATAGACGTATATATATATACAGAATTCTCTATCCCATAGCTTCAAAATGTTTCT
Donor-*TEF1p*-5'*RIM15*-R	ATGCCAAGTCCCTCTTTCATAGCCTGAGATCCGCCTGCGGTGTTACTTCTATTGAACATCTTAGATTAGATTGCTATGC
Verify-*TEF1p*-F	CGATGACCTCCCATTGATAT
Verify-5'*RIM15*-R	CTTGAACGTTATAGTGCAAC
RT-*RGS2*-F	ATTAGCGAGGAGAAGCTCTT
RT-*RGS2*-R	GCACTTCTCAAATATCTCCC
RT-*SIP18*-F	TGATTCCCATCAGAAGGGAA
RT-*SIP18*-R	TGATCATGGCCCATCTTCAT
RT-*SRX1*-F	GCTAGCAAGACATGCTCTCT
RT-*SRX1*-R	GCCGCCGAAGGCATAATATA
RT-*YDJ1*-F	TGTCACGGTACTGGTGATAT
RT-*YDJ1*-R	GACCATCTTTCATACCTGGT
gRNA-*yap1*Δ-F	CCGCAGTGAAAGATAAATGATCTGAACCAGGTATGTGGAACAGTTTTAGAGCTAGAAATAGCAAG
gRNA-*yap1*Δ-R	CTTGCTATTTCTAGCTCTAAAACTGTTCCACATACCTGGTTCAGATCATTTATCTTTCACTGCGG
Verify-gRNA-*yap1*Δ-R	TGTTCCACATACCTGGTTCA
Donor-*yap1*Δ-F1	TATACGAAGATCGGGGCTTT
Donor-*yap1*Δ-R1	CTTTAAACGTTTTGGGTGGC
Donor-*yap1*Δ-F2	GCCACCCAAAACGTTTAAAGGCGGGAACTTTATGGAAAAC
Donor-*yap1*Δ-R2	CTACTCATGCCACTAACAAG
pGADT7-scaffold-F	ATCCATCGAGCTCGAGCTGC
pGADT7-scaffold-R	GAATTCACTGGCCTCCATGGCC

Table S3. (Continued) Primers used in this study

Primers	Sequence (5'-3')
pGBKT7-scaffold-F	ATGGCCATGGAGGCCGAATT
pGBKT7-scaffold-R	CAGGTCCTCCTCGAGATCAGCTTC
pA-Yap1-F	GGCCATGGAGGCCAGTGAATTCATGAGTGTGTCTACCGCCAA
pA-Yap1-R	GCAGCTCGAGCTCGATGGATTTAGTTCATATGCTTATTCA
pB-Rim15$^{1-344}$-F	ATCTCAGAGGAGGACCTGATGTTCAATAGAAGTAACACCGC
pB-Rim15$^{1-344}$-R	AATTCGGCCTCCATGGCCATACCATTATCTTGAGGAAATGTTGA
pB-Rim15$^{359-1771}$-F	ATCTCAGAGGAGGACCTGGCTTTGTTAGATCAATTCCCTATCC
pB-Rim15$^{359-1771}$-R	AATTCGGCCTCCATGGCCATTCAGTGCGTTTCATCAGAATC
T7p-F	TAATACGACTCACTATAGG
Verify-Yap1-F	GGAAGGCTCTTTACTAAGGT
Verify-Rim15$^{1-344}$-F	TTTGCGAGCATAGAACGGAA
Verify-Rim15$^{359-1771}$-F	TGCCGGTGATGAACTAGTT
Verify-*ADH1t*-R	ATTTTCGTTTTAAAACCTAAGAGTCAC
gRNA-2716*RIM15*-F	CCGCAGTGAAAGATAAATGATCGTGAATGATATGCATCAAAAGTTTT AGAGCTAGAAATAGCAAG
gRNA-2716*RIM15*-R	CTTGCTATTTCTAGCTCTAAAACTTTTGATGCATATCATTCACGATCAT TTATCTTTCACTGCGG
Verify-gRNA-2716*RIM15*-R	TTTTGATGCATATCATTCAC
Donor-Rim15^{K823A}-F1	AGTATTTCCTTGACCCCAAG
Donor-Rim15^{K823A}-R1	CGGATTTGACATTTGTTACTTGATTTTTGGCAATCATATCTGATTTCCT TAGAACCGCTATAGCAAAATAATCTCCT
Donor-Rim15-K823A-F2	AGTAACAAATGTCAAATCCG
Donor-Rim15-K823A-R2/ Donor-Rim15-D918A-R1	CATGATGAATGATCCCGTTCTGGTGCATGTCGTTTACACCGACAACG ATTTCGGTTA
Donor-Rim15-K823A-R3	ACATGACCTGCATTATCAATTAGTAGATTTTCAGGCTTTAAGTCATGA TGAATGATCCC
Donor-Rim15-D918A-F	GCAAAGGTGCTTATGGTAGT
Donor-Rim15-D918A-R2	CCGAAATCTGTTAATTTCACATGACCTGCATTATCAATTAGTAGATTT TCAGGCTTTAAGGCATGATGAATGATCC

Table S3. (Continued) Primers used in this study

Primers	Sequence (5’-3’)
Verify-Rim15-pointmutant-R	CTTAGCGACGACTTATGTGG
Donor-Rim15-KDΔ-F1	GGCTGGGGATAAGATCAAGA
Donor-Rim15-KDΔ-R1	ATCTTTTATGCTAGGCGTTGG
Donor-Rim15-KDΔ-F2	CCAACGCCTAGCATAAAAGATAAGAATGTGGATTGGGATCATG
Donor-Rim15-KDΔ-R2	TACTTGATCTGCGATCCCTC

*Dashed underlines, cohesive terminus; Solid underlines, homologous fragment.

Table S4. Function annotation and changes of transcription level of the selected genes affected by *RIM15* deletion*.

**Genes**	**$\log_2$ (Fold change: *rim15*Δ/BY4741)**		**Description**
	Transcriptome	RT-qRCR	
ADA2	-1.26	-1.11	Transcription coactivator
AQR1	-1.29	-0.91	Plasma membrane transporter of the major facilitator superfamily
ERG5	-0.99	0.08	C-22 sterol desaturase
GND2	-1.68	-0.90	6-phosphogluconate dehydrogenase
GRE1	-1.29	-1.60	Hydrophilin essential in desiccation-rehydration process
IME2	1.12	-0.44	Serine/threonine protein kinase involved in activation of meiosis
IRA2	1.02	0.45	GTPase-activating protein
NAT4	-1.10	-0.75	N alpha-acetyl-transferase
RGS2	-1.56	-0.67	Negative regulator of glucose-induced cAMP signaling
RPI1	-1.25	-0.64	Transcription factor
SDP1	-1.58	-1.71	Stress-inducible dual-specificity MAP kinase phosphatase
SIP18	-2.82	-1.39	Phospholipid-binding hydrophilin
SKS1	-1.02	-0.77	Putative serine/threonine protein kinase
SRX1	-1.08	-2.14	Sulfiredoxin
SSA1	-1.03	-1.15	ATPase involved in protein folding and NLS-directed nuclear transport
TRA1	1.07	1.65	Subunit of SAGA and NuA4 histone acetyltransferase complexes
YDJ1	-1.24	-1.95	Type I HSP40 co-chaperone
YPK2	1.12	1.70	AGC-type S/T protein kinase

*Differential transcription levels from the transcriptome data were shown, which were further validated by RT-qPCR. Annotation of the functions is retrieved from SGD (www.yeastgenome.org.).

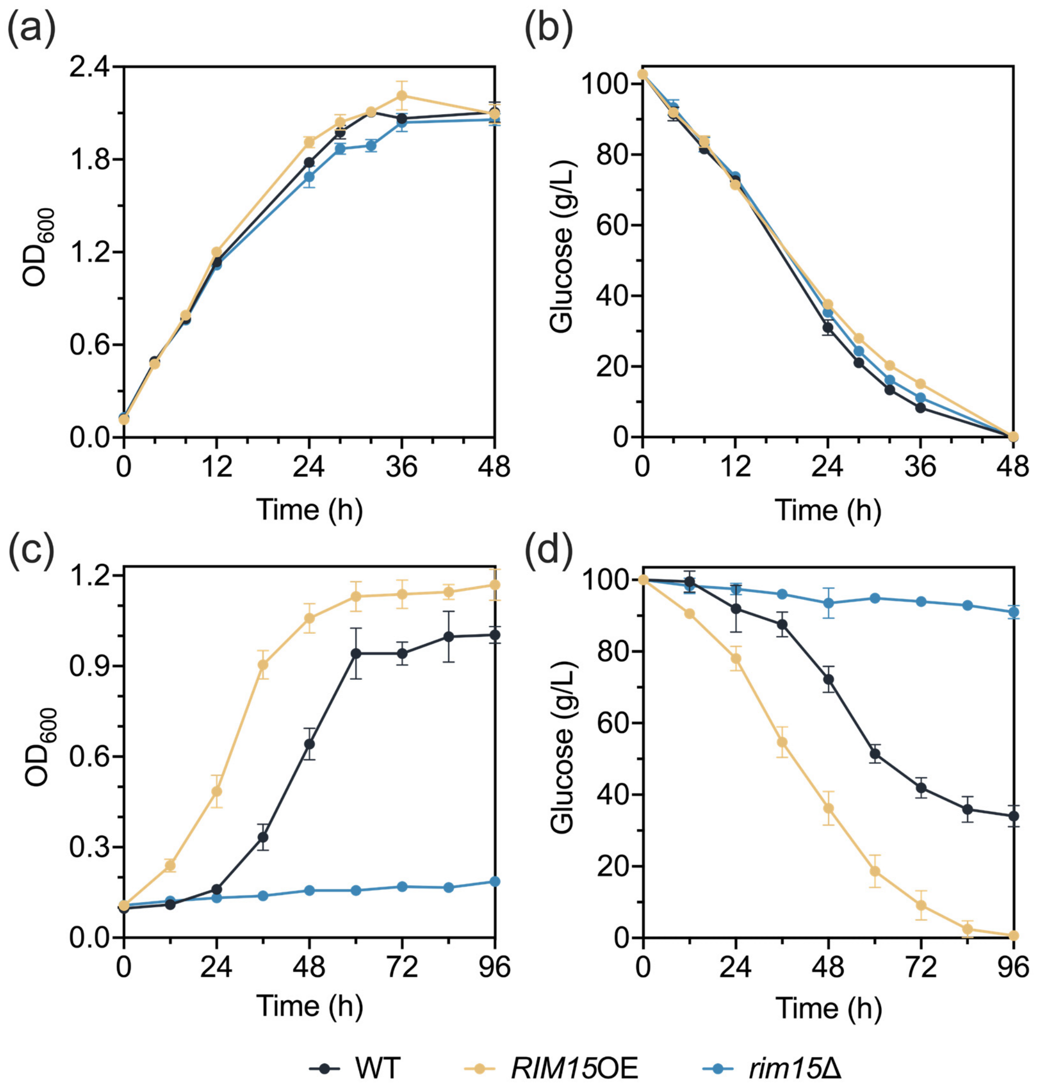

Figure S1. Effects of *RIM15* overexpression and deletion on yeast stress tolerance and fermentation performance. Fermentation of the *RIM15* overexpression strain *S. cerevisiae RIM15*OE and the *RIM15* deletion strain *S. cerevisiae rim15*Δ was performed under non-stress (**a,b**) and 5 g/L acetic acid stress (**c,d**) conditions, with *S. cerevisiae* BY4741 as a control strain. The biological triplicates were employed. Error bars represented the standard deviations.

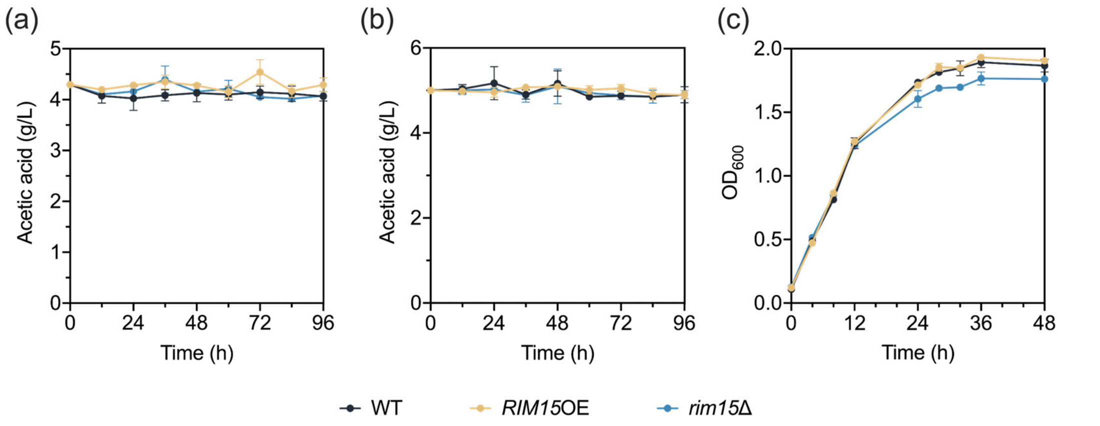

Figure S2. Effects of *RIM15* overexpression and deletion on acetic acid concentration in the fermentation broth and low pH stress tolerance. Acetic acid consumption was detected in the presence of 4.2 g/L (**a**) and 5 g/L (**b**) acetic acid. (**c**), Growth of *S. cerevisiae RIM15*OE, *rim15*Δ, and BY4741 strains was evaluated under pH3.5. The biological triplicates were employed. Error bars represented the standard deviations.

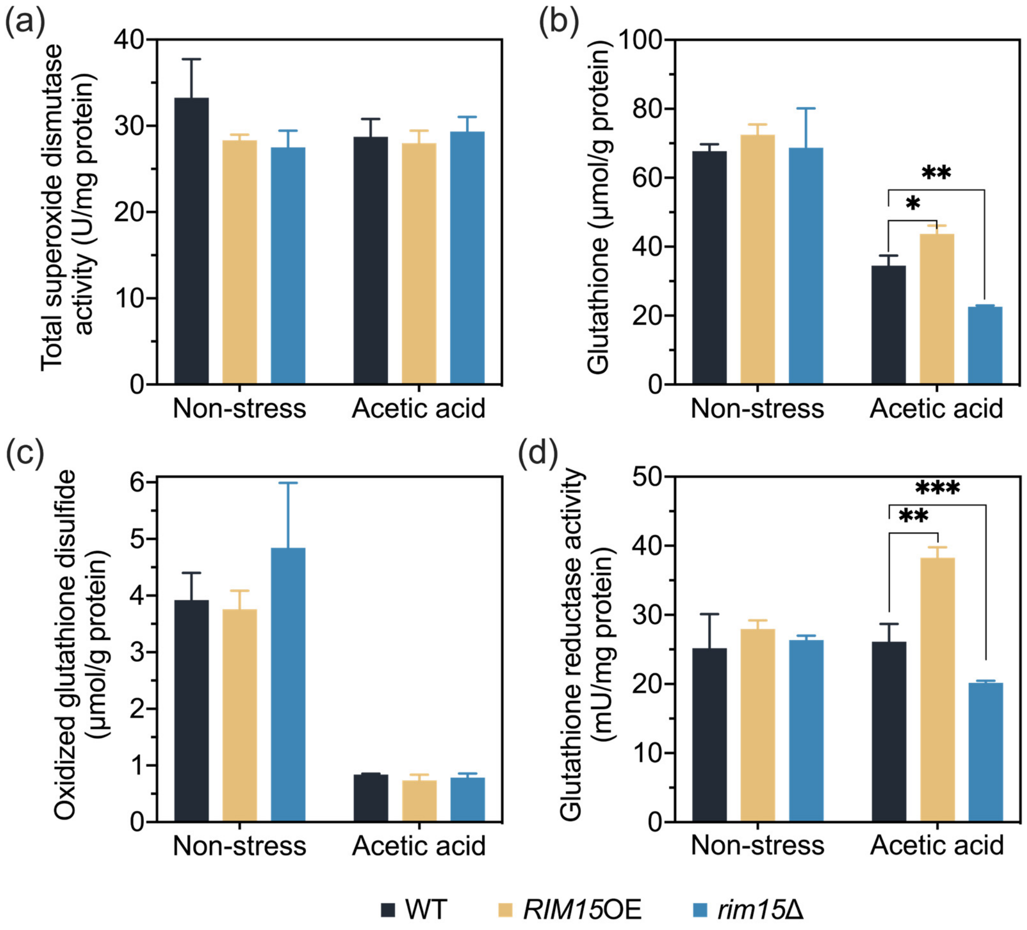

Figure S3. Effects of *RIM15* overexpression and deletion on the activities of the antioxidant system under acetic acid stress condition. SOD activity (**a**), GSH content (**b**), GSSG content (**c**), and GR activity (**d**) of *S. cerevisiae RIM15*OE, *rim15*Δ, and the control strain BY4741 were measured with or without the treatment with 4.2 g/L acetic acid. The biological triplicates were employed. Error bars represented the standard deviations. Statistical analysis was performed by *t* test, and the significant levels were indicated as follows: $*P<0.05$, $**P<0.01$, $***P<0.001$.

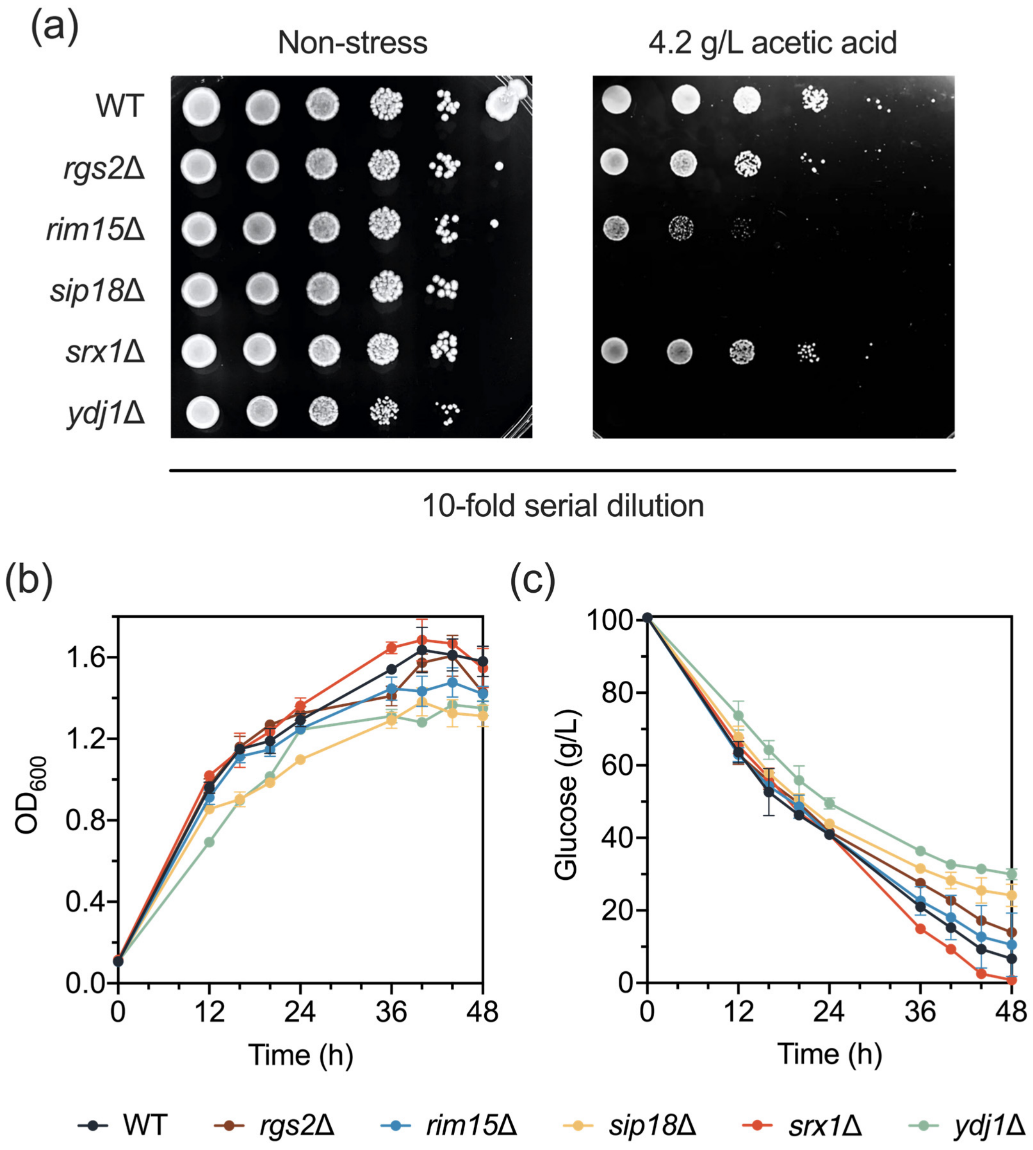

Figure S4. Evaluation of stress tolerance and fermentation performance of the gene deletion yeast strains. (**a**), Evaluation of stress tolerance with *S. cerevisiae* BY4741 and the knock-out strains of *S. cerevisiae rgs2*Δ, *rim15*Δ, *sip18*Δ, *srx1*Δ, and *ydj1*Δ was performed using spot assays under non-stress and 4.2 g/L acetic acid stress conditions. (**b,c**), Growth and fermentation performance of the yeast strains under non-stress condition. The biological triplicates were employed. Error bars represented the standard deviations.

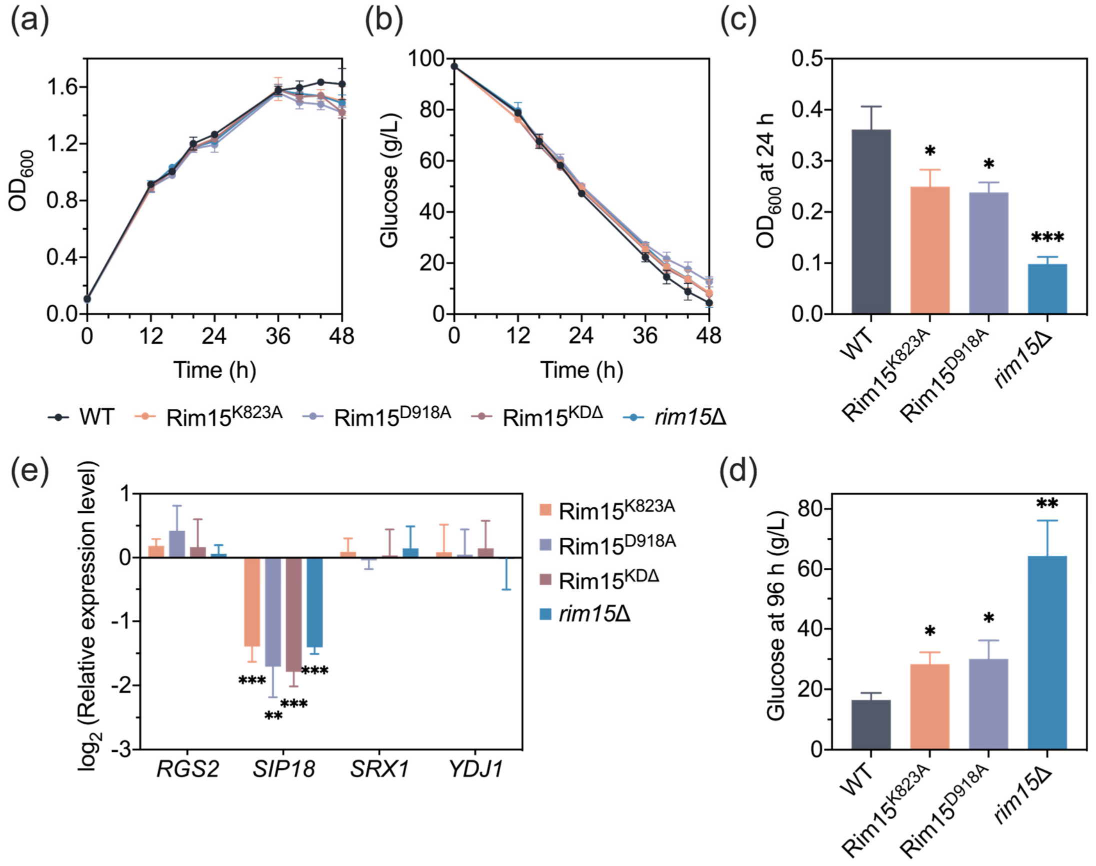

Figure S5. Effect of Rim15 kinase activity on fermentation and expression of target genes. Fermentation of *S. cerevisiae* BY4741, Rim15 kinase-dead yeast strains, and knock-out strain *rim15*Δ was evaluated under non-stress (**a, b**) and 4.2 g/L acetic acid stress (**c, d**) conditions. (**e**), Transcription of target genes *RGS2*, *SIP18*, *SRX1*, and *YDJ1* in Rim15 kinase-dead strains (Rim15^{K823A}, Rim15D^{918A}, and Rim15$^{KD\Delta}$) and knock-out strain *rim15*Δ was detected by RT-qPCR analysis without inhibitors. The biological triplicates were employed. Error bars represented the standard deviations. Statistical analysis was performed by *t* test, ∗*P*<0.05, ∗∗*P*<0.01, ∗∗∗*P*<0.001.

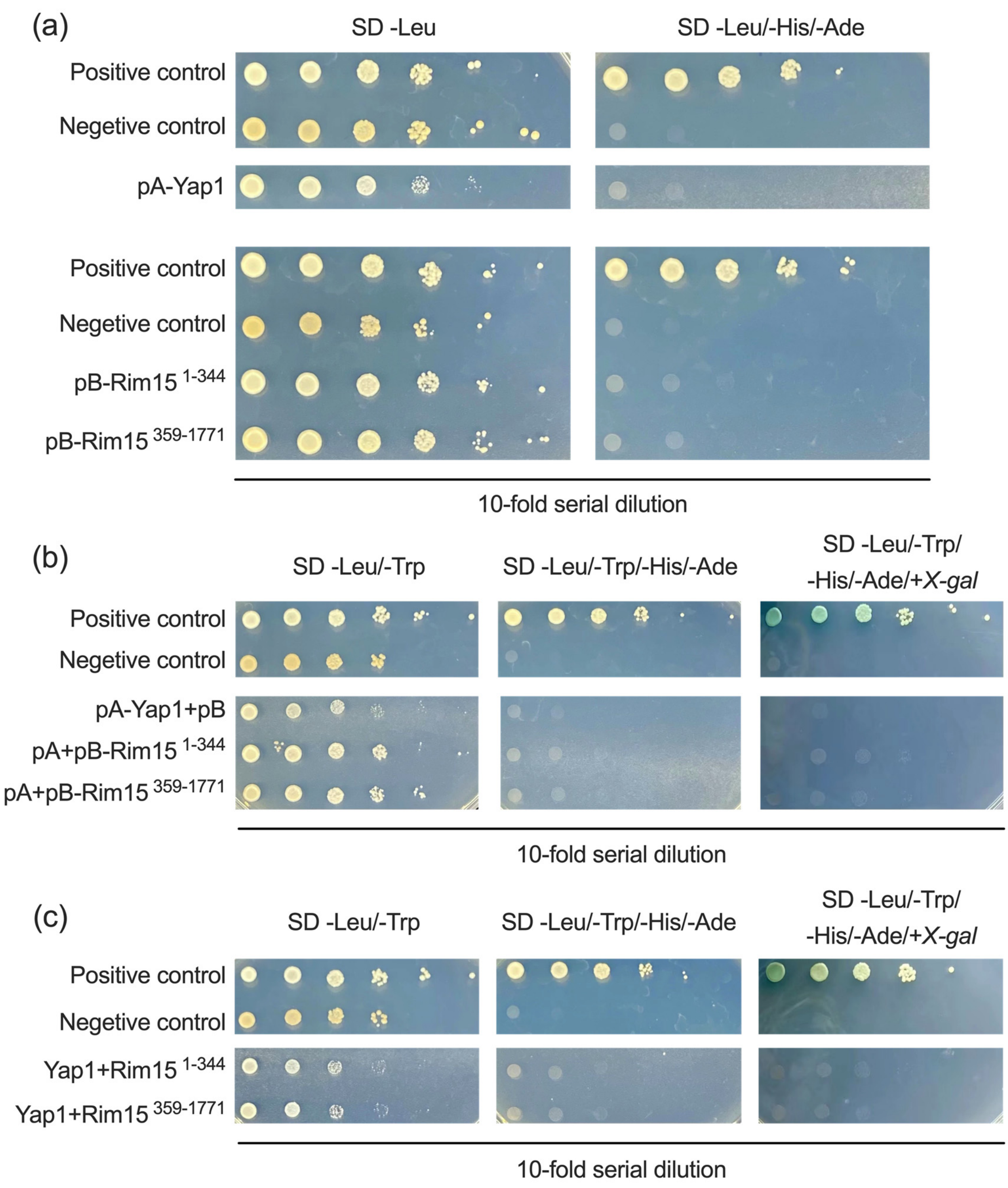

Figure S6. Analysis of protein-protein interaction of Rim15 and Yap1. The self-activation of a single protein (**a**) and protein with empty vector (**b**) was verified on SD plates without His and Ade and with *X-gal* overlay. If the colony survived on the SD agar plates lacking His and Ade or turned blue with the supplement of *X-gal*, indicating the existence of self-activation. (**c**), Yeast two-hybrid assays were employed to verify the interaction between Rim15 and Yap1 *in vivo*. If the colony survived on the SD agar plates lacking Leu, Trp, His, and Ade, or turned blue with the supplement of X-gal, indicating there was an interaction between the two proteins.

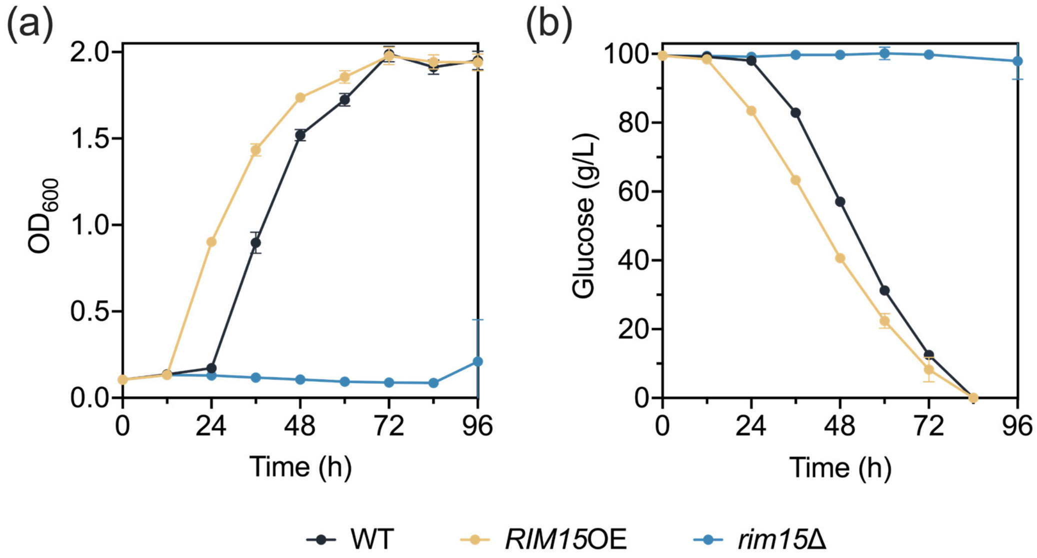

Figure S7. Effect of *RIM15* overexpression and deletion on oxidative stress tolerance and fermentation performance. Growth (**a**) and glucose consumption (**b**) of *S. cerevisiae* BY4741, *RIM15*OE, and *rim15*Δ strains were observed in the presence of 10 mM H_2O_2. The biological triplicates were employed. Error bars represented the standard deviations.

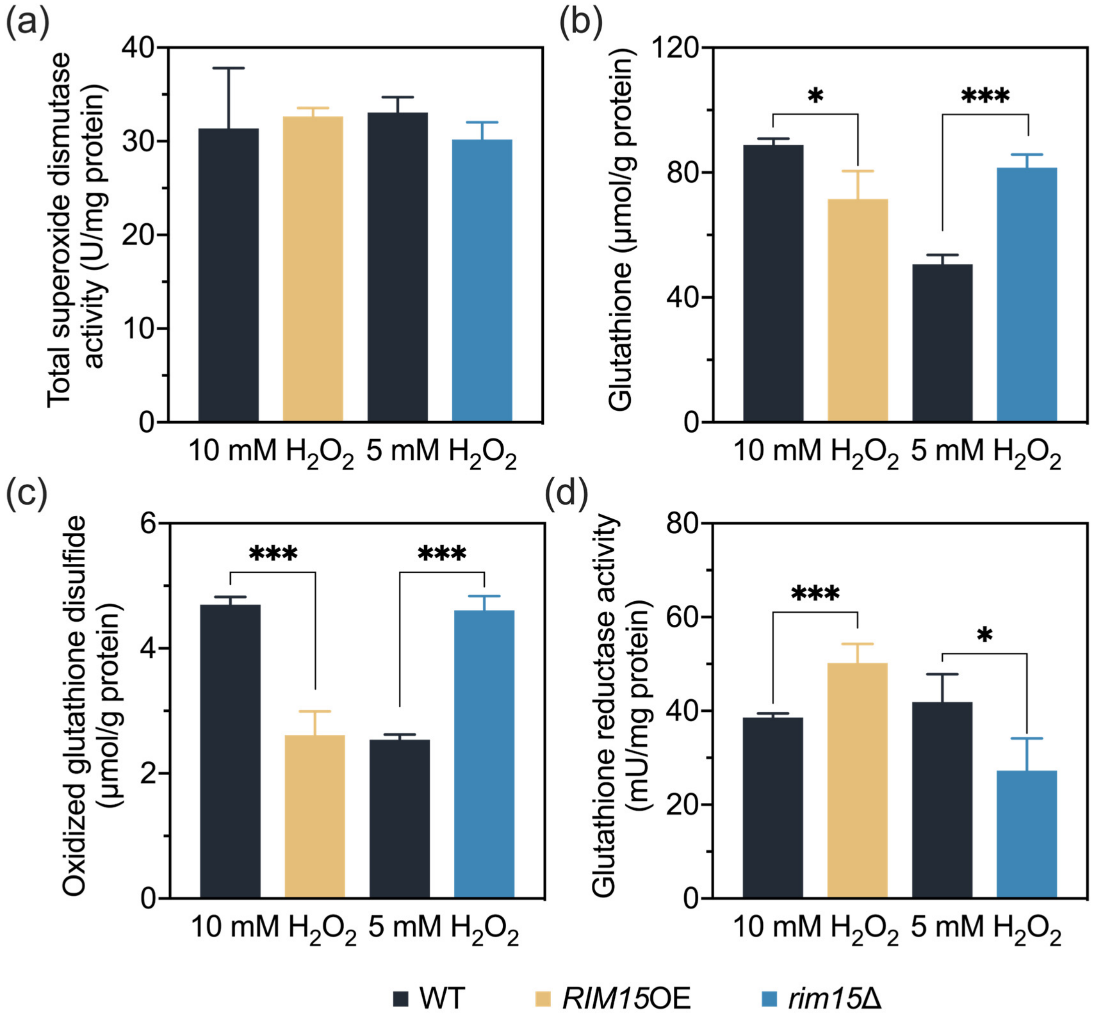

Figure S8. Effects of *RIM15* overexpression and deletion on antioxidant status of the yeast strains under oxidative stress. SOD activity (**a**), GSH (**b**) and GSSG (**c**) contents, and GR activity (**d**) of *S. cerevisiae RIM15*OE, *rim15*Δ, and BY4741 strains were measured with or without the treatment with H_2O_2. The biological triplicates were employed. Error bars represented the standard deviations. Statistical analysis was performed by *t* test, and the significant levels were indicated as follows: $*P<0.05$, $***P<0.001$.

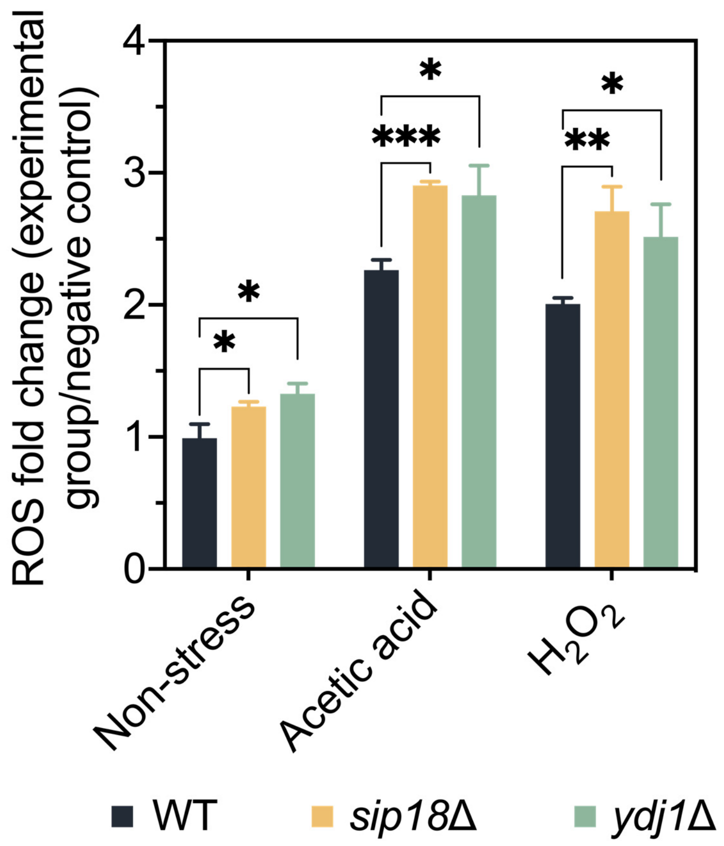

Figure S9. Effect of *SIP18* and *YDJ1* gene deletion on ROS accumulation. ROS accumulation of *S. cerevisiae* BY4741, *sip18*Δ, and *ydj1*Δ was measured under non-stress, 4.2 g/L acetic acid stress, and 5 mM H_2O_2 stress conditions, respectively. The biological triplicates were employed in experiments. Error bars represented the standard deviations. Statistical analysis was performed by *t* test, and the significant levels were indicated as follows: $*P<0.05$, $**P<0.01$, $***P<0.001$.

Reference

38. Zhang, G.C.; Kong, I.I.; Kim, H.; Liu, J.J.; Cate, J.H.; Jin, Y.S. Construction of a quadruple auxotrophic mutant of an industrial polyploid *saccharomyces cerevisiae* strain by using RNA-guided Cas9 nuclease. *Appl. Environ. Microbiol.* **2014**, *80*, 7694–7701. https://doi.org/10.1128/aem.02310-14.
